# The Role of Early Risk Factor Modification and Ablation in Atrial Fibrillation Substrate Remodeling Prevention

**DOI:** 10.3390/biomedicines13020405

**Published:** 2025-02-07

**Authors:** Ioanna Koniari, Maria Bozika, Kassiani-Maria Nastouli, Dimitra Tzegka, Anastasios Apostolos, Dimitrios Velissaris, Georgios Leventopoulos, Angelos Perperis, Nicholas G. Kounis, Grigorios Tsigkas, Periklis Davlouros

**Affiliations:** 1 Department of Medicine, Division of Cardiology, University Hospital of Patras, 265 04 Patras, Greece; mariabozika29@gmail.com (M.B.); kassienmarie@gmail.com (K.-M.N.); dimitratzeg@gmail.com (D.T.); levent2669@gmail.com (G.L.); angelosperperis@msn.com (A.P.); ngkounis@otenet.gr (N.G.K.); gregtsig@upatras.gr (G.T.); pdav@upatras.gr (P.D.); 2 Liverpool Centre for Cardiovascular Science, Liverpool L14 3PE, UK; 3 First Department of Cardiology, Hippocration General Hospital, National and Kapodistrian University of Athens, 157 72 Athens, Greece; anastasisapostolos@gmail.com; 4 Department of Internal Medicine, University Hospital of Patras, 265 04 Patras, Greece; dimitrisvelissaris@yahoo.com

**Keywords:** atrial fibrillation, risk factor modification, catheter ablation, paroxysmal atrial fibrillation

## Abstract

Atrial fibrillation (AF) is the most common sustained arrhythmia, contributing to significant morbidity and healthcare burden worldwide. This review evaluates the role of early risk factor modification and timely catheter ablation in preventing AF progression and improving patient outcomes. A comprehensive literature search was conducted using PubMed, MEDLINE, and Google Scholar, focusing on studies published after the ESC 2020 guidelines for the diagnosis and management of AF up to the release of the updated ESC 2024 guidelines for the management of AF. Keywords included “atrial fibrillation”, “catheter ablation”, “risk factor management”, and “psychological stress”. Relevant clinical trials, randomized controlled trials, systematic reviews, and meta-analyses were included, with particular emphasis on novel studies contributing to the ESC 2024 updated recommendations. Traditional risk factors such as obesity, hypertension, diabetes, sleep apnea, alcohol consumption, and physical exertion are well established in AF progression. Early evidence also suggests a role for psychological stress and mood disorders, including depression and post-traumatic stress disorder (PTSD), in increasing AF susceptibility. Psychological stress and mood disorders are linked to AF primarily through behavioral changes such as poor medication adherence, unhealthy lifestyle choices, and increased substance use. Recent guidelines recommend early catheter ablation in selected patients to reduce AF burden, prevent atrial remodeling, and improve quality of life, particularly in those resistant to antiarrhythmic drugs or individuals with AF-induced cardiomyopathy. Furthermore, we highlight the importance of a patient-centered, multidisciplinary approach, integrating electrophysiologists, cardiologists, and primary care providers with structured risk factor interventions and shared decision-making. Despite these advances, gaps remain in defining optimal timing, patient selection, and long-term benefits of catheter ablation in persistent AF, necessitating the need for further research. By integrating early intervention, personalized treatment strategies, and collaborative care models, we may usher in a paradigm shift in AF management, improving long-term cardiovascular outcomes and patient quality of life.

## 1. Introduction

AF is the most common supraventricular arrhythmia. It is characterized by rapid and disorganized atrial activity which leads to an irregular ventricular response. It is associated with significant morbidity, including a five-fold increased risk of stroke and a two-fold rise in all-cause mortality [1]. The prevalence of AF is projected to grow substantially, reaching an estimated 12.1 million individuals in the United States and up to 17 million in Europe by 2030 [2].

AF is classified as paroxysmal if it terminates spontaneously or with intervention within seven days and it often lasts less than 48 h [3]. Persistent AF exceeds seven days without spontaneous termination. Long-standing persistent AF persists for over 12 months but remains amenable to rhythm control therapies in selected cases [3]. Permanent AF is defined as when rhythm control is no longer pursued, thus following a shared decision between the patient and clinician [3].

Several modifiable risk factors contribute to the development of AF. Those include hypertension, smoking/tobacco use, alcohol intake, physical exertion, diabetes mellitus type 2 (T2D), obstructive sleep apnea (OSA), obesity, stress, and depression. Stress and depression are significant predisposing factors for AF. Rosman et al. conducted a prospective cohort study of 988,090 young and middle-aged veterans, free of AF at baseline, who accessed care through the Veterans Health Administration from 2001 to 2014. Over a mean follow-up of 4.8 years, 2491 individuals developed AF. Post-traumatic stress disorder (PTSD) was associated with a significantly higher incidence of AF (*p* < 0.0001), with affected patients developing AF at a younger age (*p* = 0.004). In unadjusted models, PTSD increased AF risk by 31% (hazard ratio 1.31; 95% CI: 1.19–1.43), and this remained significant after adjusting for demographics, lifestyle, cardiovascular factors, and depression (hazard ratio 1.13; 95% CI: 1.02–1.24). This effect was consistent across sexes (*p* = 0.93). The presence of mental health disorders not only elevates AF risk but also affects the management and outcomes of the condition. Thus, it is important to integrate mental health care into AF management.

Effective management of these risk factors plays a key role in both primary and secondary prevention. Primary prevention focuses on improving cardiovascular health so that it can prevent the progression of arrhythmic substrate and clinical AF onset. Secondary prevention emphasizes reducing AF burden and recurrence. To achieve this, it is important to enhance the efficacy of antiarrhythmic drugs and catheter ablation. Addressing these modifiable factors enables upstream management, leading to a reduction in the overall AF burden and improved treatment outcomes in affected patients. This review aims to evaluate the impact of these factors on AF progression and to highlight strategies for optimizing patient care in alignment with current evidence and updated guidelines.

## 2. Modifiable Risk Factors of AF

**Figure 1:**
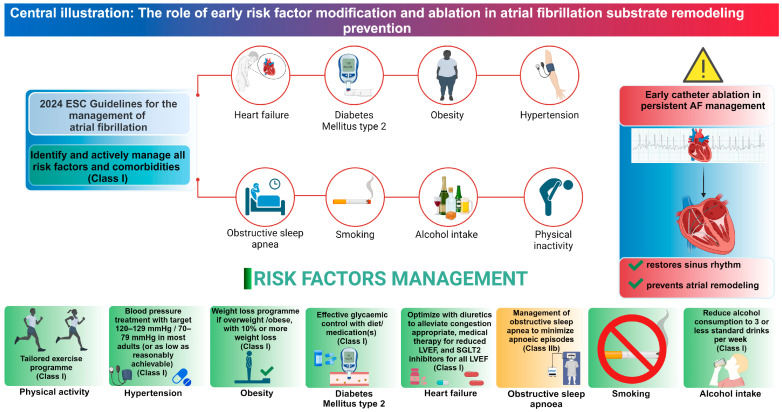
Comprehensive approach to risk factor modification and early catheter ablation in atrial fibrillation management. The figure illustrates the eight major modifiable risk factors (heart failure, diabetes mellitus type 2, obesity, hypertension, obstructive sleep apnea, smoking, alcohol intake, and physical inactivity) and their evidence-based management strategies according to the 2024 ESC Guidelines. Figure created in https://BioRender.com..

Several lifestyle and health-related factors contribute to the risk of developing AF. These include hypertension, smoking, alcohol intake, physical inactivity, obesity, diabetes mellitus type 2, obstructive sleep apnea, stress, and depression (Figure 1).

### 2.1. Alcohol Intake

Alcohol intake plays a complex role in the management and prognosis of AF. Its effects mostly depend on the quantity that is consumed. Evidence highlights a linear dose–response relationship between alcohol intake and AF risk. Even moderate consumption predisposes individuals to AF development. To clarify, moderate alcohol consumption is defined as fewer than 10 drinks per week, while heavy consumption exceeds this threshold. Larsson et al. conducted a prospective cohort study of 79,019 Swedish men and women and reported a graded increase in AF risk with alcohol intake [1]. Over a mean follow-up of 12 years, individuals consuming more than 21 drinks per week had a 39% higher risk of developing AF compared to those drinking less than one drink per week (RR: 1.39; 95% CI: 1.22–1.58). A meta-analysis within the same study confirmed that even a moderate alcohol intake of three drinks per day increased AF risk by 26% (RR: 1.26; 95% CI: 1.19–1.33) [1]. Excessive alcohol intake not only exacerbates AF incidence but also elevates the risks of adverse outcomes, including thromboembolic events, ischemic stroke, and bleeding complications [2,3]. This is particularly critical in patients receiving oral anticoagulation (OAC). In this case, alcohol can interfere with drug metabolism and adherence, as well as aggravate liver dysfunction and bleeding risks [3,4].

To address the bleeding risk in real-world AF patients, the HAS-BLED score was developed. Key risk factors such as hypertension, abnormal renal/liver function, stroke history, bleeding predisposition, and concomitant use of drugs or alcohol are incorporated [5]. This novel scoring system demonstrated strong predictive accuracy (C statistic: 0.72) and highlighted that the annual bleeding rate escalates with increasing risk factors. Alcohol consumption was identified as a significant risk factor that underlines the need for individualized bleeding risk assessment in AF patients. The HAS-BLED tool provides clinicians with a practical framework so as to balance the bleeding risks against the benefits of OAC in patients with concurrent alcohol use. Multiple studies emphasize the benefits of alcohol abstinence in improving outcomes for AF patients. Qiao et al. focused on the impact of alcohol consumption on atrial substrate remodeling and catheter ablation outcomes in 122 patients with paroxysmal AF [6]. They demonstrated that daily alcohol consumption independently predicted the presence of left atrial low-voltage zones (LVZs), a marker of atrial remodeling (OR: 1.097; 95% CI: 1.001–1.203). Furthermore, heavy drinkers revealed significantly worse ablation outcomes. Heavy drinkers presented 35.1% success rates while alcohol abstainers presented 81.3% success rates (HR: 1.579; 95% CI: 1.09–2.30). Gallagher et al. conducted a systematic review and meta-analysis that included over 12,000 AF cases. The study confirmed a dose–response relationship between alcohol intake and incidents of AF [7]. High levels of alcohol consumption were associated with a 34% increased AF risk (HR: 1.34; 95% CI: 1.20–1.49). Moderate drinking increased AF risk in men (HR: 1.26; 95% CI: 1.04–1.54) but not in women (HR: 1.03; 95% CI: 0.86–1.25), showing potential underlying gender differences in susceptibility. Voskoboinik et al. investigated the effects of alcohol abstinence in a randomized controlled trial involving 140 regular drinkers with AF [8]. The study showed that abstinence led to a 45% reduction in AF recurrence over six months compared to continued drinking (HR: 0.55; 95% CI: 0.36–0.84). Takahashi et al. evaluated the role of alcohol reduction in 1720 patients undergoing catheter ablation for AF [9]. They found that patients who reduced alcohol intake by at least 1% during a one-year follow-up had significantly lower AF recurrence rates (HR: 0.63; 95% CI: 0.52–0.77).

Recent work by Marcus et al. demonstrated the acute effects of intravenous alcohol on atrial electrophysiology properties [10]. In a double-blind, placebo-controlled trial, alcohol administration reduced atrial effective refractory periods (AERPs) and increased atrial conduction delays, both of which predispose individuals to AF onset. Lastly, Overvad et al. analyzed a Danish cohort of patients with newly diagnosed AF. In the cohort, heavy alcohol consumption was associated with a 33% increased risk of thromboembolism or death in men with an intake of more than 27 drinks per week (HR: 1.33; 95% CI: 1.08–1.63) compared to those receiving less than 14 drinks a week [4]. Additionally, Lee et al. conducted a large nationwide cohort study. They found that alcohol consumption after a new AF diagnosis significantly raised the risk of ischemic stroke. Abstinence from alcohol reduced this risk, with abstainers showing a 25% lower risk compared to current drinkers (HR: 0.75; 95% CI: 0.70–0.81) [3].

### 2.2. Type 2 Diabetes Mellitus

Diabetes mellitus (DM) is a significant and independent risk factor for cardiovascular diseases, including AF. The relationship between DM and AF is supported by structural and functional alterations in the heart [11]. Glycemic variability, oxidative stress, and chronic systemic inflammation associated with DM contribute to atrial remodeling, autonomic dysfunction, and electrical abnormalities [11]. These alterations predispose patients to the development of AF [11]. Evidence suggests that the duration of diabetes and poor glycemic control, as reflected by elevated hemoglobin A1c (HbA1c) levels, are correlated with a progressively higher risk of developing AF [12].

Dublin et al. established that pharmacologically treated DM increased the risk of developing AF by 40% (OR: 1.40; 95% CI: 1.15–1.71) in a large population-based cohort [12]. This was corroborated by Huxley et al., whose meta-analysis of over 1.6 million individuals revealed that DM was associated with a 39% increase in AF (RR 1.39,95% CI: 1.10–1.75) [13]. Building on this, the ACCORD trial, which included 10,251 patients, found that intensive glycemic control did not reduce AF incidence but was associated with increased mortality in patients with incident AF (HR: 1.22; 95% CI: 1.01–1.46). Thus, they emphasized the risks of stringent glucose-lowering strategies [14]. Chang et al. provided novel insights into metformin, demonstrating that in a cohort of 645,710 patients, its use was related to a 19% reduction in AF risk (HR: 0.81; 95% CI: 0.76–0.86), likely due to its antioxidant and antifibrotic effects on atrial tissue [15]. Chang’s subsequent study in 2017 further highlighted that dipeptidyl peptidase-4 inhibitors (DPP4is) reduced AF risk by 35% (HR: 0.65; *p* < 0.0001) in a nationwide Taiwanese cohort of 90,880 patients [16]. Similarly, Wang et al. supported the intersection of DM and AF and they identified systemic inflammation and atrial fibrosis as shared pathophysiological mechanisms [11].

The therapeutic potential of sodium–glucose cotransporter-2 (SGLT2) inhibitors has since gained prominence. Li et al. systematically reviewed 16 RCTs encompassing 38,335 patients and confirmed the efficacy of SGLT2 inhibitors in reducing AF and atrial flutter (RR: 0.76; 95% CI: 0.65–0.90) [17]. Similarly, Yin et al., through a meta-analysis of nine randomized controlled trials (10,344 patients), reported a 37% reduction in AF risk (RR: 0.63; 95% CI: 0.45–0.87) [18]. Pandey et al. demonstrated a 25% reduction in serious AF events in a pooled analysis of 75,279 participants [19]. In addition, Proietti et al. emphasized the pleiotropic benefits of SGLT2 inhibitors, including anti-inflammatory and antifibrotic effects [20]. Karamichalakis et al. [21] and Kishima et al. [22], accordingly, highlighted the utility of SGLT2 inhibitors in reducing AF recurrence in post-catheter ablation. Wang et al. expanded on this by analyzing over 52,000 patients and demonstrating an 18% reduction in AF incidence (OR: 0.82; 95% CI: 0.73–0.93) [11]. In an investigation by Lee et al., SGLT2 inhibitors were compared with DPP4 inhibitors in a cohort of over 61,000 diabetic patients, finding a reduction in new-onset AF risk (HR: 0.68; 95% CI: 0.56–0.83) [23]. Further studies reinforced the role of glycemic control in AF risk mitigation.

Tseng et al. analyzed 173,398 patients and demonstrated that metformin users had a significantly lower rate of AF-related hospitalizations compared to non-users (37.72 vs. 92.45 per 100,000 person-years; HR: 0.405; 95% CI: 0.319–0.515) [24]. Ostropolets et al. extended these findings, showing that metformin monotherapy was associated with a significantly lower risk of AF and ventricular arrhythmias compared to sulfonylureas and, as a result, emphasized metformin’s cardioprotective effects [25]. Nantsupawat et al. demonstrated that metformin reduces AF risk through AMPK-mediated suppression of inflammation and atrial fibrosis, with decreased pro-inflammatory markers including IL-6, TNF-α, TGF-β1, and NF-κΒ [26]. A landmark analysis from the DECLARE-TIMI 58 trial demonstrates that dapagliflozin reduced atrial fibrillation/flutter events by 19% (HR 0.81, 95% CI: 0.68–0.95, *p* = 0.009) in patients with type 2 diabetes mellitus. This reduction was consistent across key subgroups, including those with and without prior AF/AFL, and persisted even when excluding events associated with heart failure hospitalizations [27].

Zheng et al. conducted a systematic review and meta-analysis to investigate the relation between SGLT-2 inhibitors and the risk of occurrence of AF and stroke among a population with or without DM. Their findings highlight that SGLT2 inhibitors significantly reduced the risk of AF (OR 0.82, 95% CI: 0.72–0.93, *p* = 0.002), with dapagliflozin showing the strongest evidence for AF risk reduction (OR 0.80, 95% CI: 0.68–0.93, *p* = 0.003). However, there was no significant effect on stroke risk (OR 0.99, 95% CI: 0.85–1.15, *p*= 0.908). However, it should be taken into consideration that AF in the studies that were included was described as an adverse event and not as a primary outcome.

### 2.3. Obstructive Sleep Apnea

Obstructive sleep apnea (OSA) is a significant modifiable risk factor for AF. The mechanisms underlying this association include intermittent hypoxia, heightened sympathetic activity, and atrial remodeling. All of the aforementioned factors predispose individuals to arrhythmogenesis [28]. Continuous positive airway pressure (CPAP) therapy, a cornerstone of OSA management, has shown promise in reducing cardiovascular risk [29]. Nevertheless, its role in modifying AF burden and recurrence remains unclear [29].

Early research established a link between OSA and increased AF recurrence. Gami et al. demonstrated in their study of 3542 adults that obstructive sleep apnea and obesity independently predicted new-onset atrial fibrillation in patients under 65 years, with the magnitude of nocturnal oxygen desaturation serving as a key pathophysiological link between OSA and AF development [30]. In their study of 174 patients, Matiello et al. demonstrated that severe obstructive sleep apnea independently predicted lower success rates of atrial fibrillation ablation. Only 14.3% of severe OSA patients remained arrhythmia-free at one year compared to 48.5% in those without severe OSA (HR 1.870, *p* = 0.019) [31].

Subsequent research by Naruse et al. included 153 patients and revealed that untreated OSA increased the risk of AF recurrence by 2.6-fold following radiofrequency catheter ablation (RFCA) [32]. Importantly, CPAP use significantly attenuated this risk, with treated patients exhibiting recurrence rates comparable to those without OSA. Fein et al. evaluated 62 OSA patients undergoing AF ablation. They demonstrated that CPAP therapy significantly improved AF-free survival rates (71.9% in CPAP users vs. 36.7% in non-users, *p* = 0.01), with treated patients achieving outcomes comparable to those without OSA [33]. Subsequently, the larger SAVE trial enrolled 2717 patients with cardiovascular disease and OSA to assess CPAP’s effect on cardiovascular events [34]. Despite improving sleep apnea parameters, CPAP therapy did not significantly reduce cardiovascular events or new-onset AF during the 3.7-year follow-up. These contrasting findings suggest that CPAP has potentially targeted benefits in post-ablation AF management, while the impact on broader cardiovascular prevention remains limited.

Qureshi et al. performed a meta-analysis of eight studies encompassing 1247 patients and they revealed that CPAP therapy was associated with a 42% reduction in AF recurrence after cardioversion or catheter ablation in OSA patients [35]. Holmqvist et al. analyzed data from the Outcomes Registry for Better Informed Treatment of Atrial Fibrillation (ORBIT-AF), which included 10,132 patients, of whom 1841 had OSA. OSA patients had a higher risk of hospitalization (HR 1.12, 95% CI: 1.03–1.22) but no significant differences in death, cardiovascular events, major bleeding, or AF progression vs. those without OSA. Among OSA patients, CPAP use was associated with a lower risk of AF progression (HR 0.66, 95% CI: 0.46–0.94) [29]. A study by Christensen et al. revealed that fragmented sleep and reduced REM sleep were independently associated with elevated AF risk. Frequent nighttime awakenings conferred a 33% higher risk and each standard deviation decrease in REM sleep increased the risk by 18% [36].

Furthermore, Genuardi et al. analyzed a cohort of 30,061 individuals and linked short sleep durations to both prevalent and incident AF. They demonstrate that each hour of sleep deprivation increased the odds of prevalent AF by 17% and the risk of incident AF by 9% [37]. Hojo et al. studied 100 patients with OSA undergoing PVI and found AF recurrence rates of 12.1% in non-OSA patients, 9.1% in CPAP-treated OSA patients, and 8.7% in untreated OSA patients after the second PVI (*p* = 0.944) [38]. Hunt et al.’s randomized trial of 108 paroxysmal AF patients with moderate-to-severe OSA showed 57% AF recurrence in both CPAP and control groups 3–12 months post-PVI (OR 1.0, 95% CI: 0.4–2.4, *p* = 1.0) [39]. AF burden decreased from 5.6% to 4.1% with CPAP and 5.0% to 4.3% with usual care (adjusted difference: −0.63 percentage points; 95% CI: –2.55–1.30; *p* = 0.52). Traaen et al. conducted a randomized controlled trial involving 108 patients and found that CPAP reduced AF burden by only 0.6 percentage points more than the control (95% CI: –2.6–1.3, *p* = 0.52) [40]. Although CPAP effectively reduced apnea severity, it failed to demonstrate a significant reduction in AF burden compared to standard care.

### 2.4. Physical Exertion and Moderate Physical Activity

Physical inactivity is a critical and modifiable risk factor for atrial fibrillation. Early investigations into the relationship between physical activity and AF risk began with Hegbom et al. who conducted a randomized controlled trial in 30 chronic AF patients [41]. They demonstrated that a 2-month exercise program significantly improved quality of life and reduced symptom burden compared to controls. In 2009, Abdulla and Nielsen performed a systematic review and meta-analysis, encompassing 655 athletes and 895 controls, finding a significantly higher risk of AF in athletes (odds ratio: 5.29; 95% CI: 3.57–7.85). The metanalysis suggested a U-shaped relationship between exercise intensity and AF risk [42]. Osbak et al. further investigated this relationship through a randomized controlled trial of 49 patients with permanent AF. They revealed that 12 weeks of supervised aerobic exercise significantly improved exercise capacity (6MWT: 504.4 ± 85.1 m to 569.9 ± 92.6 m, *p* < 0.001) and reduced resting heart rate (94.8 ± 22.4 bpm to 86.3 ± 22.5 bpm, *p* = 0.049) [43]. Similarly, the CARDIO-FIT study of 308 obese AF patients demonstrated that higher cardiorespiratory fitness (CRF) was associated with a 20% reduction in AF recurrence risk per metabolic equivalent increase, while achieving a ≥2 MET gain further reduced symptom severity and AF burden [44]. Also, another randomized controlled trial (n = 51 patients) revealed that a 12-week aerobic interval training program reduced AF burden from 8.1% to 4.8% and significantly improved quality of life [45].

In a nationwide cohort study, Jin et al. analyzed 501,690 individuals, observing that moderate activity levels (500–1000 MET-minutes/week) reduced AF risk by 12% (hazard ratio: 0.88; 95% CI: 0.80–0.97), but excessive activity (≥1000 MET-minutes/week) showed no additional benefit. Thus, a U-shaped dose–response relationship was highlighted [46]. Elliott et al. examined data from 402,406 UK Biobank participants. They identified that those achieving 1500 MET-minutes/week experienced significant reductions in AF risk (hazard ratio: 0.85; 95% CI: 0.74–0.98), with women benefiting more than men [47]. Khurshid et al. enhanced the methodological rigor by analyzing accelerometer-derived physical activity data from 93,669 individuals and they confirmed that adherence to guideline-recommended activity levels (≥150 min/week) lowered AF risk (hazard ratio: 0.82; 95% CI: 0.75–0.89) and demonstrated greater accuracy than self-reported measures [48]. Mishima et al. synthesized data from over 1.4 million individuals in a systematic review, reaffirming that guideline-recommended activity levels (450 MET-minutes/week) reduced AF risk by 6% (hazard ratio: 0.94; 95% CI: 0.90–0.97) but benefits plateaued beyond 1900 MET-minutes/week [49]. In 2022, a meta-analysis of 1155 participants across 13 randomized controlled trials concluded that supervised exercise reduced AF recurrence (relative risk: 0.77; 95% CI: 0.60–0.99) and improved quality of life [50]. Finally, the ACTIVE-AF trial demonstrated that a 6-month combined supervised and home-based exercise program in 120 symptomatic AF patients reduced recurrence risk (hazard ratio: 0.50; 95% CI: 0.33–0.78) and improved symptom severity [51].

### 2.5. Obesity

Obesity is a well-established modifiable risk factor for AF and is associated with atrial structural remodeling, inflammation, and autonomic dysfunction [52,53,54]. Its management is crucial for reducing AF incidence, recurrence, and symptom burden [55]. Both surgical and nonsurgical weight reduction strategies have demonstrated significant benefits in addressing these risks [55]. The prospective cohort study by Tedrow et al. based on the Women’s Health Study followed 34,309 women over a mean period of 12.9 years, revealing a strong linear association between BMI and AF risk [56]. Each unit increase in BMI was associated with a 4.7% higher risk of AF (*p* < 0.0001), while obesity (BMI ≥ 30 kg/m^2^) increased the AF risk by 77% (HR: 1.77; 95% CI: 1.47–2.11). Short-term BMI increases also significantly elevated AF risk. This finding emphasized the importance of long-term weight stability in mitigating AF burden. A single-center randomized controlled trial evaluated the efficacy of structured weight management programs in 150 symptomatic AF patients with elevated BMI or waist circumference (BMI > 27 kg/m^2^ or a waist circumference > 100 cm for men and > 90 cm for women) [57]. Participants were randomized to receive either intensive weight management or general lifestyle advice. At a median of 15 months, patients in the intervention group had significantly greater reductions in BMI and AF symptom burden. Furthermore, the frequency and duration of AF episodes were significantly reduced in this group compared to controls (*p* < 0.001).

Lifestyle modifications have also demonstrated improved efficacy in AF management. The ARREST-AF study evaluated the effects of aggressive risk factor management (RFM) in 149 overweight and obese patients (BMI ≥ 27 kg/m^2^) who underwent catheter ablation [58]. Patients receiving RFM, which included weight loss, exercise, and management of comorbidities, exhibited higher arrhythmia-free survival rates compared to controls (45.5% vs. 18.8%, *p* < 0.001) after a single ablation procedure. Furthermore, RFM led to significant reductions in AF symptom burden and severity, a fact that demonstrated the importance of comprehensive lifestyle interventions in conjunction with procedural treatments. The LEGACY trial supported the profound benefits of sustained weight loss in AF management [59]. Among 355 overweight and obese patients, those that achieved ≥10% weight loss experienced a sixfold improvement in arrhythmia-free survival compared to <3% weight loss (*p* < 0.001). A landmark study by Jamaly et al. based on the Swedish Obese Subjects (SOS) cohort evaluated 4021 obese patients free of AF at baseline. Out of the 4021 patients, 2000 underwent bariatric surgery and 2021 received the usual care [60]. Over a median follow-up of 19 years, bariatric surgery was associated with a 29% lower risk of new-onset AF compared to controls (HR: 0.71; CI: 0.60–0.83; *p* < 0.001). This benefit was more obvious in younger patients and those with elevated diastolic blood pressure. Importantly, sustained weight loss of approximately 18% at 20 years in the surgery group contrasted with minimal changes in the control group. This outcome underlined the role of long-term weight stability in AF prevention. Long-term observational data from Bunch et al. assessed the influence of BMI on AF outcomes in 1558 patients undergoing index AF ablation [61]. A higher BMI was associated with increased AF recurrence at the 3-year follow-up (*p* = 0.02). Additionally, both the lowest and highest BMI groups experienced elevated risks of stroke and heart failure. These findings underscore the importance of addressing BMI extremes in AF management. The role of nonsurgical weight loss interventions was investigated by Mohanty et al. in a study of 90 morbidly obese patients with longstanding persistent AF [62]. Of these, 58 underwent dietary and exercise interventions over a year, while the remaining 32 served as controls. Significant weight loss was achieved in the intervention group (median −24.9 kg, *p* < 0.0001), accompanied by improvements in physical and mental quality-of-life scores. However, arrhythmia-free survival at 1 year post-ablation did not differ significantly between the two groups (63.8% vs. 59.3%, *p* = 0.68).

The EPIC-Norfolk study, a prospective cohort investigation of 21,499 participants, further underlined the importance of lifestyle behaviors, signifying that adherence to multiple healthy lifestyle factors reduced AF risk by nearly threefold compared to individuals adhering to none (HR 2.82, *p* < 0.001) [63]. The impact of bariatric surgery on AF recurrence after ablation was further explored in a retrospective cohort study involving 239 morbidly obese patients [64]. Of these, 51 underwent bariatric surgery, while 188 served as controls and were enrolled in weight management clinics. Over a mean follow-up of 36 months post-ablation, AF recurrence rates were significantly lower in the BS group (20%) compared to controls (61%, *p* < 0.0001). Repeat ablation was also less frequent in the BS group (12% vs. 41%, *p* < 0.0001). Recurrence was defined as arrhythmia episodes lasting ≥30 s, confirmed by ECG or device interrogation. Roux-en-Y gastric bypass was the predominant surgical method employed in the BS cohort. The Lynch et al. study used a propensity-matched analysis of 5044 patients (2522 surgical and 2522 nonsurgical) to evaluate the impact of bariatric surgery on AF prevention [65]. Over a median follow-up of 7 years, bariatric surgery was associated with a significantly lower incidence of new-onset AF compared to nonsurgical management (0.8% vs. 2.9%, *p* < 0.0001). Surgical patients achieved a greater reduction in excess BMI, which likely contributed to the observed cardioprotective effects. Donnellan et al. conducted a retrospective observational cohort study of 440 morbidly obese patients (BMI ≥ 40 kg/m^2^) with AF to investigate the impact of BS on AF progression [66]. Patients were matched 1:1 between BS and nonsurgical weight management groups. The follow-up extended to 12 months post-surgery. The findings revealed a significant reduction in AF-type progression in the BS group (3%) compared to controls (14%, *p* < 0.001). The AF-type reversal was exclusively observed in the surgical cohort. These benefits correlated with marked BMI reductions, from 49.7 ± 9 kg/m^2^ to 38.5 ± 9 kg/m^2^ in the BS group (*p* < 0.0001). Similarly, the BLOC-AF study evaluated the impact of bariatric surgery on the incidence of AF and heart failure hospitalizations [67]. This study analyzed data from 1581 patients who underwent bariatric surgery and were matched with 3162 controls who had other abdominal surgeries. Over a mean follow-up of 66 months, bariatric surgery was associated with a 29% reduction in the risk of first-time AF hospitalization (HR 0.71; 95% CI: 0.54–0.93) and a 26% reduction in heart failure hospitalizations (HR 0.74; 95% CI: 0.60–0.91). These results suggested that substantial weight loss can mitigate the risk of AF and its complications in obese individuals. A meta-analysis by Chokesuwattanaskul et al. assessed the incidence of AF following bariatric surgery [68]. This analysis included seven studies with a total of 7681 patients and reported a pooled AF incidence of 5.3% post-surgery. Compared to nonsurgical controls, bariatric surgery was associated with a 58% reduction in the risk of developing AF (OR 0.42; 95% CI: 0.22–0.83) over a median follow-up of 7.9 years. Additional evidence supporting the benefits of bariatric surgery includes the Höskuldsdóttir et al. study, which demonstrated a 41% reduction in AF incidence (HR 0.59; CI: 0.44–0.78) among 10,642 patients with type 2 diabetes mellitus and obesity undergoing Roux-en-Y gastric bypass [69].

### 2.6. Hypertension

Hypertension is a major risk factor for AF, contributing to structural and electrical remodeling of the atria and increasing the likelihood of recurrent episodes [70,71]. Effective management of blood pressure is essential not only for the prevention of AF but also for maintaining sinus rhythm post-cardioversion, as demonstrated in studies utilizing medications like enalapril, irbesartan, and metoprolol [72,73,74]. Research has consistently shown that renin–angiotensin–aldosterone system inhibitors and beta-blockers can reduce AF recurrence and associated complications, particularly in hypertensive populations [71,72]. Early evidence was provided by the LIFE study in 2005, which analyzed 9193 hypertensive patients with left ventricular hypertrophy [75]. This study demonstrated that losartan significantly reduced the incidence of new-onset AF compared to atenolol (6.8 vs. 10.1 per 1000 person-years, HR 0.67, *p* < 0.001). These results underlined the potential of angiotensin II receptor blockers (ARBs) to mitigate AF risk by addressing atrial remodeling. Similarly, the VALUE trial in 2008, which included 15,245 hypertensive patients, showed that valsartan reduced the incidence of new-onset AF (3.67% vs. 4.34%, HR 0.843, *p* = 0.0455) and persistent AF (1.35% vs. 1.97%, HR 0.683, *p* = 0.0046) compared to amlodipine [76].

Later, a nested case–control study by Schaer et al. analyzed 4661 patients with AF and 18,642 matched controls from a population of 682,993 hypertensive patients in the UK General Practice Research Database [77]. The study found that long-term therapy with ACE inhibitors (OR 0.75; CI: 0.65–0.87), ARBs (OR 0.71; CI: 0.57–0.89), or β-blockers (OR 0.78; CI: 0.67–0.92) was correlated with a significantly lower risk of incident AF compared to calcium-channel blockers. Further insights were provided by Okin et al. who studied 8831 hypertensive patients with left ventricular hypertrophy [78]. Lower systolic blood pressure (SBP) ≤ 130 mm Hg was associated with a 40% reduction in AF risk compared to SBP ≥ 142 mm Hg, emphasizing the importance of rigorous blood pressure control. The ACCORD BP trial also investigated intensive BP management in 3087 patients with diabetes, finding that targeting SBP < 120 mm Hg reduced a composite outcome of AF and *p*-wave abnormalities by 13% (HR 0.87, *p* = 0.02). However, its direct impact on AF alone was not statistically significant [79]. Dewland et al. revisited data from the ALLHAT trial involving 14,837 hypertensive patients without prevalent AF or atrial flutter [80]. According to the study’s outcomes, neither lisinopril (HR 1.04; CI: 0.94–1.15) nor amlodipine (HR 0.93; CI: 0.84–1.03) was found to decrease the risk of incident AF or flutter compared to chlorthalidone during a mean follow-up of 7.5 years. These findings highlighted the lack of consistent evidence supporting the use of these agents for primary AF prevention in older adults.

In contrast, the SPRINT trial reported a 26% reduction in new-onset AF with intensive BP control among 8022 hypertensive patients without diabetes (HR 0.74, *p* = 0.037), supporting its effectiveness in non-diabetic populations [81]. A meta-analysis by Neefs et al. explored the impact of mineralocorticoid receptor antagonists (MRAs) and revealed a 52% reduction in new-onset AF risk (OR 0.52, *p* < 0.001) and a 57.9% reduction in AF recurrence (OR 0.37, *p* < 0.001). The study suggested the utility of MRAs in preventing atrial fibrosis [82]. Similarly, Schneider et al. combined data from 23 trials involving 87,048 patients, concluding that RAS inhibitors reduced the odds of AF by 33% in primary prevention and 45% in secondary prevention (*p* < 0.00001) [83]. More recent evidence by Pinho-Gomes et al. analyzed data from 188,570 patients across 22 trials and demonstrated that a 5 mm Hg reduction in SBP significantly lowered cardiovascular event risk in patients with AF (HR 0.91, *p* = 0.037). [84]. However, Parkash et al., in a study of 184 patients undergoing catheter ablation, reported no significant difference in AF recurrence between aggressive BP control (<120/80 mm Hg) and standard BP control, though a potential benefit was noted in older patients [85].

### 2.7. Smoking/Tobacco Use

Tobacco smoking is a well-established modifiable risk factor for cardiovascular diseases and plays a significant role in the development and progression of atrial fibrillation. It promotes atrial remodeling through oxidative stress, inflammation, and fibrosis, creating a substrate conducive to AF initiation and persistence [86,87,88]. Components of cigarette smoke, including nicotine and carbon monoxide (CO), contribute to these changes [89]. Nicotine induces fibrosis via transforming growth factor-beta (TGF-β) pathways and suppresses protective microRNAs, while CO exacerbates tissue hypoxia, enhancing arrhythmogenic remodeling [89]. Oxidative stress further damages cardiac tissues, amplifying structural and electrical instability. Together, these effects highlight the multifaceted impact of smoking on AF progression [89].

Early investigations, such as the study by Fukamizu et al., explored the effect of cigarette smoking on AF recurrence after pulmonary vein isolation [90]. This study of 59 patients revealed that smokers experienced significantly higher recurrence rates (43%) compared to non-smokers (14%). The relative risk of AF recurrence among smokers was 3.19 and, thus, the role of smoking in exacerbating atrial substrates through fibrotic remodeling was highlighted. The Atherosclerosis Risk in Communities (ARIC) study provided a broader epidemiological perspective [91]. This large cohort study of over 15,000 participants examined the association between smoking and incident AF. The results revealed a clear dose–response relationship, with current smokers having a hazard ratio of 2.05 for AF compared to non-smokers and former smokers also displaying an elevated risk (HR 1.32). Accumulated smoking exposure significantly amplified AF risk, confirming the negative impact of smoking on atrial remodeling and arrhythmogenesis. Albertsen et al. investigated the effect of smoking on thromboembolism and mortality in AF patients using data from a large Danish cohort [92]. Among 3161 patients with incident AF, heavy smoking was associated with a substantially increased risk of thromboembolism or death. The adjusted HR was 3.64 for women and 2.17 for men, emphasizing the severe implications of smoking in the context of AF, particularly among female patients.

Subsequent efforts to synthesize evidence led to a systematic review and meta-analysis by Aune et al. [93]. This analysis included data from 29 prospective studies, revealing that current smokers had a 32% increased risk of AF compared to non-smokers. The analysis also demonstrated a clear dose–response relationship, with a 14% increased risk per 10 cigarettes smoked daily, solidifying smoking as a modifiable risk factor for AF in a dose-dependent manner. Further insights into the interplay between smoking and AF outcomes were provided by Cheng et al. [94]. This study assessed the long-term effects of smoking on catheter ablation outcomes in patients with persistent AF. Smokers were found to have a higher incidence of non-pulmonary vein triggers and worse long-term outcomes, especially in cases involving right atrial triggers. Choi et al. examined the impact of smoking cessation on cardiovascular outcomes in South Korean men diagnosed with AF [95]. The study demonstrated that smoking cessation after an AF diagnosis reduced cardiovascular disease and stroke risks by 35% to 50% compared to continued smoking individuals. Most recently, Lin et al. investigated substance use, including tobacco, and its correlation with incident AF in a large-scale longitudinal study [96]. Tobacco smoking was associated with an HR of 1.35 for incident AF and its role as a preventable risk factor was reinforced.

### 2.8. Heart Failure

Heart failure (HF) is a critical determinant of prognosis in AF, with bidirectional interactions influenced by shared risk factors such as hypertension, diabetes, and aging. Insights from the Framingham Heart Study reveal that 57% of patients with HF develop AF, while 37% of AF cases occur in those with HF [97]. AF exacerbates HF through irregular ventricular rates and reduced cardiac output. HF predisposes individuals to AF via structural remodeling and neurohormonal changes [98]. Patients with both conditions face a two-fold increase in stroke risk, even with anticoagulation, and a 25% higher mortality rate [99]. Mortality is highest in those with a reduced ejection fraction (HFrEF), although stroke and hospitalization rates are similar across HF subtypes [100]. Echocardiography adds prognostic value by identifying severe left ventricular dysfunction as a key stroke risk factor [101]. Modifiable risks like obesity and hypertension further predict AF progression [102]. Effective management of AF in HF integrates pharmacological interventions, such as beta-blockers and RAAS inhibitors, alongside lifestyle changes [98,99].

The evidence on the role of ARBs in AF prevention among HF patients is conflicting. The ACTIVE I study showed that irbesartan did not significantly reduce AF recurrence or cardiovascular events, despite modest reductions in blood pressure, suggesting limited benefit in AF prevention [103]. Conversely, the CHARM program found that candesartan improved HF outcomes, including cardiovascular mortality and hospitalizations, but its direct effect on AF prevention was not definitive [104]. Kotecha et al. conducted a meta-analysis on beta-blocker efficacy in patients with HF and AF. Their study revealed a lack of clear benefits for AF-related outcomes [105]. In contrast to sinus rhythm patients, where beta-blockers reduced all-cause mortality and hospitalizations, no significant impact was observed in the AF group for mortality or hospitalizations. The RATE-AF randomized trial compared low-dose digoxin and bisoprolol for rate control in patients with permanent AF and HF [106]. While no significant difference in quality of life was observed at six months between digoxin and bisoprolol groups (adjusted mean difference in SF-36 PCS: 1.4 [95% CI: −1.1–3.8; *p* = 0.28]), digoxin demonstrated several favorable outcomes in the overall study population at 12 months, including greater symptom improvement with 68.5% achieving a two-class improvement in modified EHRA functional status versus 29.2% with bisoprolol (adjusted odds ratio 5.3 [95% CI: 2.5–11.3]; *p* < 0.001) and lower NT-proBNP levels (median 960 pg/mL vs. 1250 pg/mL; ratio of geometric means 0.77 [95% CI: 0.64–0.92; *p* = 0.005]). Additionally, digoxin showed a better safety profile with fewer adverse events (25% vs. 64% of patients) while achieving similar heart rate control. Similarly, the RACE II study demonstrated that lenient and strict rate control approaches have comparable outcomes in quality of life and cardiovascular morbidity, reinforcing the importance of tailoring therapy [107]. A meta-analysis by Ziff et al. highlights that digoxin demonstrates a neutral effect on all-cause mortality in randomized trials, with a statistically significant reduction in hospital admissions across various study types [108]. Despite observational data linking digoxin to increased mortality, these findings are largely due to confounding factors and prescription bias rather than a direct causal relationship. Importantly, the meta-regression revealed that higher-quality studies and those with lower bias more consistently showed neutral or beneficial effects on outcomes, emphasizing the need for randomized trials in guiding treatment decisions.

Both beta-blockers and digoxin offer benefits beyond prognosis: beta-blockers reduce hospitalizations and mortality in sinus rhythm HF patients, while digoxin aids in rate control and symptom relief in AF. When used appropriately, both drugs maintain a favorable safety profile for managing coexisting AF and HF [109]. Recent advances in HFrEF management have validated therapies such as eplerenone, sacubitril–valsartan, and SGLT2 inhibitors in patients with AF. The EMPHASIS-HF trial demonstrated eplerenone’s ability to reduce cardiovascular mortality and hospitalizations without efficacy differences between AF and non-AF subgroups [110]. Similarly, the PARADIGM-HF trial confirmed the positive effects of sacubitril–valsartan on mortality and hospitalizations irrespective of AF status [111]. A meta-analysis of SGLT2 inhibitors, involving over 75,000 participants across 31 randomized controlled trials, confirmed consistent reductions in cardiovascular death, HF hospitalizations, and serious AF events, highlighting their efficacy in HFrEF management regardless of AF presence [19]. Cardiac resynchronization therapy (CRT) has been thoroughly detailed in the 2021 ESC Guidelines on cardiac pacing and resynchronization therapy [112]. These guidelines emphasize the importance of ensuring effective biventricular pacing for optimal outcomes, particularly in patients with HFrEF and atrial fibrillation. A key recommendation includes a low threshold for considering atrioventricular node ablation to maintain pacing efficacy and reduce arrhythmic burden. Additionally, CRT strategies have expanded to include promising modalities such as conduction system pacing, aiming to enhance synchronization and improve patient outcomes.

In patients with HFmrEF and AF, treatment often aligns with strategies established for HFrEF [112], although evidence specific to this subgroup remains limited. Findings from the CHARM study revealed that candesartan significantly reduced cardiovascular death and HF hospitalizations in HFmrEF, similar to its effects in HFrEF. [113]. The TOPCAT trial indicated that spironolactone may benefit HFmrEF patients, particularly those with lower ejection fractions [114]. Cleland et al. found that beta-blockers improved ejection fractions and reduced mortality in HFmrEF patients with sinus rhythm, but had less benefit in those with AF [115]. For HFpEF patients with AF, SGLT2 inhibitors have demonstrated efficacy. The EMPEROR-Preserved trial reported that empagliflozin reduced cardiovascular death or HF hospitalization by 21%, with consistent benefits regardless of AF status [116]. Similarly, the SOLOIST-WHF trial confirmed sotagliflozin’s effectiveness in reducing total HF events (hospitalizations and urgent visits), including in patients with preserved EF [117]. Additionally, the DELIVER trial found that dapagliflozin significantly lowered cardiovascular death or worsening of HF risk in HFpEF, independent of AF status [118].

The Routine versus Aggressive upstream rhythm Control for prevention of Early AF in heart failure (RACE 3) trial investigated the impact of combined management strategies on sinus rhythm maintenance in patients with persistent atrial fibrillation and mild-to-moderate heart failure [119]. At one year, targeted therapy involving ACE inhibitors/ARBs, mineralocorticoid receptor antagonists, statins, and cardiac rehabilitation improved sinus rhythm maintenance compared to conventional therapy. However, a follow-up at five years revealed no sustained advantage in sinus rhythm maintenance or cardiovascular outcomes [120].

### 2.9. Depression and Stress

Although not yet incorporated into the latest ESC Guidelines on AF management, mental health disorders have been associated with AF. Disorders such as depression, anxiety, and insomnia have been associated with a higher incidence of AF, particularly in patients with comorbid conditions like diabetes [121]. These disorders may influence AF risk through mechanisms including autonomic nervous system dysregulation, inflammatory processes, and activation of the renin–angiotensin–aldosterone system, all of which create an arrhythmogenic environment in the atria [121]. Contrary to common assumptions, individuals without mental disorders sometimes exhibit higher rates of smoking and alcohol consumption, though patients with mental health conditions often display a greater prevalence of cardiovascular comorbidities like hypertension and dyslipidemia, which can contribute to AF development [121]. Furthermore, certain psychotropic medications, especially antipsychotics, are known to increase AF risk due to their effects on autonomic regulation and arrhythmogenesis [122].

In a prospective cohort study from the Multi-Ethnic Study of Atherosclerosis (MESA), 6644 middle- and older-aged adults, free of AF at baseline, were followed over a median period of nearly 13 years [123]. The study found that individuals with depressive symptoms, defined by a Center for Epidemiologic Studies Depression Scale (CES-D) score ≥ 16, had a 34% increased risk of developing AF (HR 1.34; 95% CI: 1.04–1.74). Similarly, the use of antidepressants was associated with a 36% higher risk of AF (HR 1.36; 95% CI: 1.04–1.77). In contrast, no significant associations were observed between anger, anxiety, or chronic stress and the development of AF [123]. Kim et al. conducted a cohort study that included 5031,022 individuals, of whom 148,882 had depression before the study initiation [124]. The analysis revealed that patients with depression showed a higher cumulative incidence of AF (4.44%) compared to those without depression (1.92%). Patients with depression were associated with a 25.1% increased risk for AF after adjusting for covariates (HR 1.25; 95% CI: 1.22–1.29) [124]. In the population with recurrent episodes of depression, an even higher risk of AF incidence was reported (HR 1.32; 95% CI: 1.27–1.37). Subgroup analyses highlighted strong associations in younger patients aged 20–39 years (HR 1.58; 95% CI: 1.24–2.02) and women (HR 1.32; 95% CI: 1.27–1.37) compared to men (HR 1.17; 95% CI: 1.12–1.22) [124]. These findings, based on over 43 million person-years of follow-up, suggest the need for enhanced AF screening in patients with depression, particularly among younger individuals and women [124]. Ahn et al. conducted a nationwide, population-based cohort study and analyzed data from 6576,582 young adults (aged 20–39) in Korea, free of AF at baseline, to assess the association between mental disorders and incident AF [125]. Over the follow-up period, 8932 new AF cases were recorded. Mental disorders, including depression, insomnia, anxiety, bipolar disorder, and schizophrenia, were linked to a significantly higher AF risk (adjusted HR 1.526; 95% CI: 1.436–1.621). Specifically, bipolar disorder and schizophrenia were associated with a 2-fold increase in AF risk, while depression, insomnia, and anxiety showed a 1.5- to 1.7-fold increase compared to individuals without mental disorders [125]. Thrall et al. conducted a prospective study and assessed the prevalence and persistence of depression and anxiety in 101 patients with AF compared to 97 hypertensive patients in sinus rhythm [126]. Over a 6-month follow-up, 38% of AF patients exhibited symptoms of depression and 37.6% showed elevated trait anxiety at baseline, with symptoms persisting in 36.8% and 33.3%, respectively. The study showed that depression was the strongest independent predictor of reduced quality of life (QoL) at 6 months (*p* < 0.001). A population-based cohort study followed 114,337 Danish adults from the Danish National Health Survey (2010) over 4 years to assess whether perceived stress increases the risk of AF [127]. Perceived stress was measured using Cohen’s 10-item Perceived Stress Scale (PSS). While individuals in the highest stress quintile initially showed a 28% higher AF risk compared to the lowest quintile (95% CI: 12–46%), this association disappeared after adjusting for comorbidities, lifestyle factors, and socioeconomic status (adjusted HR 1.01; 95% CI: 0.88–1.16) [127]. A meta-analysis evaluated the impact of anxiety on AF recurrence following catheter ablation. The study pooled data from five cohort studies involving 549 AF patients who underwent catheter ablation [128]. Over a mean follow-up of 9.7 months, 39.3% experienced AF recurrence. Anxiety was independently associated with a significantly higher risk of AF recurrence in post-catheter ablation (adjusted relative risk [RR]: 2.36; 95% CI: 1.71–3.26; *p* < 0.001).

A population-based study (the HUNT study) followed 37,402 Norwegian adults from 2006 to 2015 to investigate the association between anxiety, depression, and AF [129]. Over a median follow-up of 8.1 years, 1433 participants (3.8%) developed AF. While mild-to-moderate depression was linked to an increased AF risk (HR 1.5; 95% CI: 1.2–1.8), severe depression (HR 0.9; 95% CI: 0.6–1.3) and anxiety symptoms (HR 1.0–1.1) showed no significant association with AF. A nationwide cohort study investigated disparities in OAC initiation among 673 Danish patients with schizophrenia and AF compared to matched AF patients without schizophrenia [130]. Over a 1-year follow-up, only 33.7% of patients with schizophrenia initiated OAC, compared to 54.4% without schizophrenia (adjusted risk difference: −19.4%; 95% CI: −23.6 to −15.3). Although OAC initiation improved over time, particularly after the introduction of non-vitamin K antagonist oral anticoagulants (NOACs), significant disparities persisted. Fenger-Grøn et al. examined oral anticoagulation therapy use in 147,810 Danish patients with incident AF including 1208 with bipolar disorder and 572 with schizophrenia [131]. Both conditions were associated with significantly lower OAT initiation within 90 days post-AF diagnosis (bipolar disorder: −12.7%, 95% CI: −15.3%–−10.0%; schizophrenia: −24.5%, 95% CI: −28.3%–−20.7%). Even after adjusting for socioeconomic factors and comorbidities, patients with schizophrenia exhibited persistent treatment disparities (adjusted difference: −15.5%; 95% CI: −19.3%–−11.7%). Wu et al. conducted a systematic review and meta-analysis and assessed the relationship between psychological factors and AF risk, analyzing data from 13 cohort studies involving 5329,908 participants [132]. The study found that anxiety increased AF risk by 10% (HR 1.10; 95% CI: 1.02–1.19), anger by 15% (HR 1.15; 95% CI: 1.04–1.26), depression by 25% (HR 1.25; 95% CI: 1.12–1.39), and work stress by 18% (HR 1.18; 95% CI: 1.05–1.32). The study suggests that preventing mental health disorders could help reduce the global burden of AF and its healthcare costs.

A nationwide nested case–control study examined the association between antipsychotic use and AF risk using data from the Taiwan National Health Insurance Database [133]. The study included 34,053 AF cases and 34,919 matched controls from 2001 to 2010. Current antipsychotic use was linked to a 17% increased AF risk compared to non-users (adjusted OR: 1.17; 95% CI: 1.10–1.26), with a clear dose-dependent relationship (*p* for trend < 0.001). Antipsychotics with higher muscarinic M2 receptor binding affinity showed stronger associations with AF risk, particularly in patients with hypertension, diabetes, or coronary artery disease.

A prospective cohort study utilized data from the Swedish Longitudinal Occupational Survey of Health (SLOSH), including 13,200 working adults, to examine the association between job strain (a measure of work stress) and AF. Over a median follow-up of 5.7 years, 145 incident AF cases were identified. Job strain was associated with an almost 50% increased risk of AF (HR 1.48; 95% CI: 1.00–2.18), independent of lifestyle and health-related confounders. A meta-analysis combining this study with two previous ones yielded a pooled hazard ratio of 1.37 (95% CI: 1.13–1.67), emphasizing work stress as a potential AF risk factor [134].

## 3. Discussion

Effective management of modifiable risk factors is essential for preventing AF and sustaining sinus rhythm. Table 1 provides a comprehensive overview of key clinical studies that showed significant improvements in AF-related endpoints through targeted interventions, such as weight loss programs, exercise regimens, and aggressive risk factor management [109,122].

**Figure 2:**
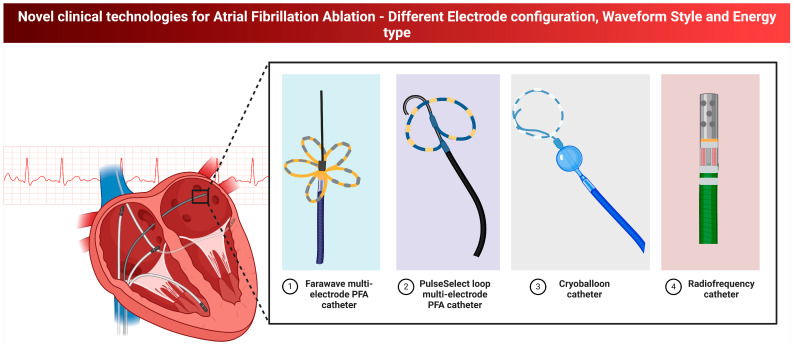
Novel clinical technologies for atrial fibrillation ablation—different electrode configurations, waveform styles, and energy types. The image illustrates the following catheters: (1) Farawave multi-electrode PFA catheter, (2) PulseSelect loop multi-electrode PFA catheter, (3) cryoballoon catheter, and (4) radiofrequency catheter. Figure created with Biorender.com.

Catheter ablation is an effective treatment option for reducing AF recurrences and improving AF burden and also quality of life in symptomatic patients with paroxysmal or persistent AF who are either intolerant to or unresponsive to antiarrhythmic drug (AAD) therapy [135,136,137,138,139,140,141]. Guidelines recommend catheter ablation in these patients to reduce symptoms, recurrence, and progression of AF (Class I, Level A) [135,137,139,140,141]. While the CABANA trial did not demonstrate clear mortality or stroke reduction benefits compared to medical therapy, its results were likely affected by high crossover rates and low event rates [138]. Nonetheless, CABANA highlighted significant reductions in AF burden and improvements in quality of life for patients undergoing ablation. Cryoballoon ablation offers comparable efficacy to radiofrequency ablation, with shorter procedural times and similar safety profiles. Novel technologies such as Pulsed Field Ablation (PFA) have also demonstrated promising results (Figure 2). For rhythm control in paroxysmal AF, catheter ablation is strongly recommended as a first-line therapy (Class I, Level A) [135,137,139,140,142]. For persistent AF, its use as a first-line option is reserved for carefully selected patients in the context of shared decision-making (Class IIb, Level C) [109]. The CASTLE-AF trial further highlighted the value of catheter ablation in patients with HF and reduced ejection fraction, showing significant reductions in all-cause mortality and HF hospitalizations (HR: 0.62; *p* = 0.007) compared to medical therapy [143]. Additionally, it demonstrated improvements in left ventricular ejection fraction and reduced cardiovascular deaths, underscoring the importance of sinus rhythm restoration in this subgroup.

Supported by strong evidence and guideline recommendations, catheter ablation offers a patient-centered approach to reducing symptoms, improving rhythm outcomes, and enhancing quality of life in AF management. The treatment choice in everyday clinical practice relies on shared decision-making. For the improvement of symptoms in patients, even in persistent AF, operators proceed with catheter ablation because sinus rhythm establishment is crucial and inhibits atrial remodeling. This dual focus on symptomatic relief and long-term cardiac health brings out the key role of catheter ablation in current AF management.

**Table 1:** Summary of key studies investigating the impact of modifiable risk factor management on atrial fibrillation outcomes.

Author/Study Design	Population	Sample Size	Variable	Endpoint	Outcome	Follow-Up
Alcohol Intake						
Overvad et al./prospective cohort study (2013) [4]	Participants with incident AF from the Danish Diet, Cancer, and Health study	3107	Alcohol consumption	Thromboembolism or death	Men with an alcohol intake of more than 27 drinks per week had a higher risk of thromboembolism or death, with a hazard ratio (HR) of 1.33 (95% CI: 1.08–1.63). In women, those with an intake of more than 20 drinks per week had a higher risk with an HR of 1.23 (95% CI: 0.78–1.96). The higher risk in men was primarily driven by mortality (HR 1.51, 95% CI: 1.20–1.89), while in women, the higher risk was driven by thromboembolism (HR 1.71; 95% CI: 0.81–3.60).	Median follow-up of 4.9 years for men and 4.7 years for women
Larsson et al./prospective cohort study (2014) [1]	Swedish men and women free from AF at baseline	79,019	Alcohol consumption	Incidence of AF	Increased risk of AF with each 1 drink/day increment in alcohol consumption. The RR for men was 1.08 (95% CI: 1.06–1.11), while for women, it was 1.08 (95% CI: 1.03–1.13), and the combined meta-analysis RR was 1.08 (95% CI: 1.06–1.10).	12 years
Pathak et al./cohort study of risk factor management (RFM) and AF ablation outcomes (ARREST-AF cohort) (2014) [58]	Patients who underwent AF ablation, with a body mass index (BMI) ≥ 27 and at least one cardiac risk factor	149	Alcohol consumption among other factors such as BMI, hypertension, and diabetes	Arrhythmia-free survival after ablation	The RFM group showed significantly better outcomes with greater reductions in weight, blood pressure, glycemic control, and lipid profiles compared to the control group. The single-procedure drug-unassisted arrhythmia-free survival rate was significantly higher in the RFM group (*p* < 0.001), and the multiple-procedure arrhythmia-free survival rate was also better in the RFM group (*p* < 0.001). The risk factor management group had a hazard ratio of 4.8 (95% CI: 2.04–11.4, *p* < 0.001) for improved arrhythmia-free survival.	Mean follow-up duration of 41.6 months in the RFM group and 42.1 months in the control group
Qiao et al./single-center, observational study (2015) [6]	Patients with PAF undergoing CPVI categorized into 3 groups (abstainers, moderate drinkers, and heavy drinkers)	122	Alcohol consumption	Presence of low-voltage zones	Higher AF recurrence in heavy drinkers (35.1%) vs. moderate drinkers (69.2%) and abstainers (81.3%), *p* < 0.001. Alcohol consumption predicted low-voltage zones (OR 1.097, *p* = 0.047).	20.9 ± 5.9 months
Gallagher et al./systematic review and meta-analysis (2017) [7]	Seven studies with a total of 249,496 participants	249,496	Alcohol consumption	Incidence of AF	High levels of alcohol intake were associated with an increased risk of incident AF with a hazard ratio (HR) of 1.34 (95% CI: 1.20–1.49, *p* < 0.001). Moderate alcohol intake was associated with a small increase in AF risk in males (HR 1.26; 95% CI: 1.04–1.54; *p* = 0.02), but not in females (HR 1.03; 95% CI: 0.86–1.25; *p* = 0.74). Low alcohol intake up to 1 drink/day was not associated with an increased risk of AF (HR 0.95; 95% CI: 0.85–1.06; *p* = 0.37).	Varies
Voskoboinik et al./open-label, multicenter, randomized controlled trial (2020) [8]	Patients with symptomatic AF consuming ≥ 10 drinks/week randomized to abstinence or continued consumption	140	Alcohol abstinence	AF burden and recurrence at 6 months	Abstinence reduced AF burden by 42% (*p* < 0.01); AF recurrence in abstainers 53% vs. drinkers 73%. Benefits more pronounced in paroxysmal AF patients.	6 months
Lim et al./prospective observational registry (2021) [2]	Nonvalvular AF patients categorized by alcohol consumption levels (light, moderate, or heavy)	9411	Alcohol consumption	Composite of thromboembolism and hospitalization	Higher event rate in heavy drinkers (HR 1.32; 95% CI: 1.06–1.66; *p* = 0.02) compared to abstainers/rare drinkers. Moderate and light drinkers showed no increased risk.	17.4 ± 7.3 months
Lee et al./nationwide, population-based cohort study (2021) [3]	Comparison of drinkers, abstainers, and non-drinkers after AF diagnosis	97,869	Alcohol consumption	Risk of ischemic stroke	Stroke incidence lower in abstainers (IR 8.0/1000 PY) vs. drinkers (IR 10.0/1000 PY), *p* < 0.001. Risk reduction most significant in those ceasing alcohol post-diagnosis.	5 years
Marcus et al./randomized, double-blind, placebo-controlled trial (2021) [10]	Patients undergoing PVI randomized to alcohol infusion or placebo	100	Alcohol infusion during PVI	Changes in atrial effective refractory period	Decrease in AERP by 12 ms in alcohol group (*p* = 0.026); no significant change in placebo (*p* = 0.98). Larger proportion of decreased AERP sites in alcohol group (*p* = 0.0043). More sustained AF episodes in placebo group after isoproterenol (*p* = 0.027).	During procedure
Takahashi et al./multicenter prospective observational study (2021) [9]	Patients undergoing AF ablation encouraged to reduce alcohol consumption	1720	Alcohol consumption reduction	AF/atrial tachycardia recurrence-free survival	Reduced alcohol intake linked to lower AF recurrence risk (HR 0.63; 95% CI: 0.52–0.77; *p* < 0.001). Median alcohol reduction: 75%.	1 year
Type 2 Diabetes Mellitus						
Dublin et al./population-based case–control study (2010) [12]	Newly recognized AF cases and matched controls examining diabetes duration and glycemic control	3613	Type 2 diabetes mellitus	Risk of AF in terms of diabetes duration and glycemic control	Each year of diabetes duration increased AF risk by 3% (95% CI: 1–6%). Poor glycemic control (HbA1c > 9) associated with higher AF risk (OR 1.96; 95% CI: 1.22–3.14).	Varies
Huxley et al./systematic review and meta-analysis (2011) [13]	Meta-analysis of cohort and case–control studies examining the association of DM with AF risk	1686,097	Type 2 diabetes mellitus	Association between DM and AF risk	Patients with DM had a 40% greater risk of AF (RR 1.39; 95% CI: 1.10–1.75; *p* < 0.001). Adjusted estimates: RR 1.24 (95% CI: 1.06–1.44).	Varies
Chang et al./population-based cohort study (2014) [15]	Patients with DM using metformin vs. non-users in Taiwan National Health Insurance Database	645,710	Metformin use	AF risk reduction and oxidative stress markers	Metformin reduced AF risk (HR 0.81; 95% CI: 0.76–0.86; *p* < 0.0001) and decreased oxidative stress in atrial cells.	13 years
Fatemi et al./randomized controlled trial (2014) [14]	Patients with DM from ACCORD cohort	10,082	Intensive glycemic control	Incident AF and cardiovascular outcomes	Intensive glycemic control did not affect incident AF rate (*p* = 0.52). Incident AF associated with higher all-cause mortality (HR 2.65; 95% CI: 1.8–3.86; *p* < 0.0001) and increased risk of heart failure (HR 3.80; 95% CI: 2.48–5.84; *p* < 0.0001).	Median 4.68 years
Chang et al./nationwide cohort study using Taiwan’s National Health Insurance Research Database (2017) [16]	Patients with T2DM prescribed metformin as the first-line hypoglycemic agent	90,880 patients (16,017 using DPP-4 inhibitors and 74,863 using other hypoglycemic agents)	DPP4i usage versus other hypoglycemic agents	Risk of new-onset AF	DPP4i users had a significantly lower risk of new-onset AF compared to non-DPP4i users. Hazard ratio (HR): 0.65 (95% CI: 0.56–0.76, *p* < 0.0001). Multivariate analysis confirmed lower AF risk (HR: 0.69; 95% CI 0.59–0.81; *p* < 0.0001). Age > 65 years, hypertension, and ischemic heart disease were independent risk factors for new-onset AF.	Over a 3-year period
Zelniker et al./post hoc analysis of DECLARE-TIMI 58 trial (2020) [27]	T2DM patients with/without a history of AF or cardiovascular disease randomized to dapagliflozin or placebo	17,160	SGLT2 inhibitor (dapagliflozin)	AF/AFL events	Dapagliflozin reduced AF/AFL events by 19% (HR 0.81; 95% CI: 0.68–0.95; *p* = 0.009); consistent effects across subgroups.	Median 4.2 years
Ostropolets et al./observational retrospective cohort study (2021) [25]	Patients with type 2 diabetes mellitus using metformin compared with those using sulfonylureas, DPP4 inhibitors, thiazolidinediones, and GLP-1 receptor agonists as monotherapy	190,180 patients on metformin, 241,917 on sulfonylureas, 99,050 on DPP4 inhibitors, 88,258 on thiazolidinediones, and 26,380 on GLP-1 receptor agonists	Metformin vs. other antidiabetic drugs	The study measured the occurrence of various arrhythmias, using diagnosis codes in patient records to identify these events	The use of metformin was associated with a significantly lower risk of atrial fibrillation (HR 0.84; 95% CI: 0.81–0.88; *p* < 0.01), bradycardia (HR 0.83; 95% CI: 0.79–0.88; *p* < 0.01), and atrial flutter or supraventricular arrhythmia (HR 0.84; 95% CI 0.81–0.86; *p* < 0.01) compared to sulfonylureas. A 34% reduction in the risk of ventricular tachycardia or fibrillation was also observed compared to sulfonylureas (HR 0.66; 95% CI: 0.47–0.91; *p* = 0.01). Metformin showed consistent risk reductions compared to DPP4 inhibitors and thiazolidinediones for atrial arrhythmias but not for ventricular arrhythmias. There was no significant difference in risk compared to GLP-1 receptor agonists. Combination therapy with metformin and sulfonylureas was associated with an increased risk of atrial arrhythmias compared to metformin with DPP4 inhibitors.	Varies
Tseng/retrospective cohort study (2021) [24]	T2DM patients on metformin vs. non-users from Taiwan National Health Insurance database	195,064	Metformin use	Hospitalization for AF	Metformin users had a 40% lower risk of hospitalization for AF (HR 0.617; 95% CI: 0.441–0.864; *p* < 0.05).	6 years
Kishima et al./randomized controlled trial (2022) [22]	AF patients with DM undergoing catheter ablation randomized to SGLT2 inhibitors vs. DPP4 inhibitors	80	SGLT2 inhibitors	AF recurrence after ablation	SGLT2 inhibitors reduced AF recurrence compared to DPP4 inhibitors (24% vs. 47%; *p* = 0.0417).	1 year
Wang et al./systematic review and meta-analysis (2022) [144]	RCTs comparing SGLT2 inhibitors vs. placebo in T2DM/heart failure patients	52,951	SGLT2 inhibitors	AF/AFL incidence	SGLT2 inhibitors reduced AF/AFL incidence by 18% (OR 0.82; 95% CI: 0.73–0.93; *p* = 0.002).	Varies
Yin et al./meta-analysis of RCTs (2022) [18]	HF patients with/without T2DM receiving SGLT2 inhibitors vs. placebo	10,344	SGLT2 inhibitors	Cardiac arrhythmias (AF/AFL)	SGLT2 inhibitors reduced AF risk by 37% (RR 0.63; 95% CI: 0.45–0.87; *p* < 0.05); AF/AFL risk by 34% (RR 0.66; 95% CI: 0.49–0.90; *p* < 0.05).	Varies
Lee et al./population-based cohort study (2023) [23]	Type 2 DM patients treated with SGLT2 vs. DPP4 inhibitors	72,746	SGLT2 vs. DPP4 inhibitors	Risk of AF and cardiovascular outcomes	SGLT2 inhibitors reduced AF risk (HR 0.68; 95% CI: 0.56–0.83; *p* = 0.0001) and cardiovascular mortality (HR 0.39; 95% CI: 0.27–0.56; *p* < 0.0001).	2030 days
Obstructive Sleep Apnea						
Gami et al./retrospective cohort study (2007) [30]	Adults referred for initial diagnostic polysomnography	3542	Obstructive sleep apnea	Incident atrial fibrillation	OSA predicted new-onset AF (HR 2.18; 95% CI: 1.34–3.54). Obesity and nocturnal oxygen desaturation also independently increased AF risk.	Mean 4.7 years
Matiello et al./prospective observational study (2010) [31]	AF patients stratified by OSA severity (low risk, non-severe, or severe)	174	Obstructive sleep apnea	AF recurrence post-ablation	Severe OSA was an independent predictor of ablation failure (HR 1.87; 95% CI: 1.11–3.16; *p* = 0.019).	Varies
Naruse et al./prospective observational study (2013) [32]	Patients with drug-refractory AF undergoing PVI	153	OSA and CPAP therapy	AF recurrence after PVI	Untreated OSA doubled AF recurrence risk (HR 2.61; 95% CI: 1.12–6.09, *p* < 0.05). CPAP use halved recurrence risk (HR 0.41; 95% CI: 0.22–0.76; *p* < 0.01).	18.8 ± 10.3 months
Fein et al./retrospective observational study (2013) [33]	OSA patients with AF undergoing PVI categorized into CPAP users vs. non-users	426 (62 with OSA)	CPAP therapy	Arrhythmia-free survival post-PVI	CPAP users had higher arrhythmia-free survival (71.9% vs. 36.7%, *p* = 0.01). CPAP reduced AF recurrence risk (HR 0.7, *p* = 0.39).	12 months
Holmqvist et al./registry-based observational study (ORBIT-AF) (2015) [29]	Outpatients with AF with/without OSA	10,132	Obstructive sleep apnea	Progression of AF and hospitalizations	OSA increased hospitalization risk (HR 1.12; 95% CI: 1.03–1.22; *p* = 0.0078). CPAP use reduced progression to permanent AF (HR 0.66; 95% CI: 0.46–0.94; *p* = 0.021).	Up to 2 years
Qureshi et al./systematic review and meta-analysis (2015) [35]	OSA patients undergoing CPAP therapy	698 CPAP users; 549 non-users	CPAP therapy	Recurrence of AF	CPAP use reduced AF recurrence by 42% (RR 0.58; 95% CI: 0.47–0.70; *p* < 0.001). Benefits stronger in younger, obese males.	Varies
McEvoy et al./randomized controlled trial (SAVE) (2016) [34]	OSA patients with coronary or cerebrovascular disease	2717	CPAP therapy	Major cardiovascular events	CPAP did not reduce cardiovascular event rates (HR 1.10; 95% CI: 0.91–1.32; *p* = 0.34) but improved sleep quality and reduced snoring.	Mean 3.7 years
Christensen et al./multi-cohort analysis (2018) [36]	Participants from Health eHeart Study, Cardiovascular Health Study, and California HCUP	Combined > 14 million individuals	Sleep disruption (insomnia and reduced REM sleep)	Prevalence and incidence of AF	Frequent nighttime awakening increased AF risk by 33% (HR 1.33; 95% CI: 1.17–1.51; *p* < 0.001); insomnia diagnosis increased AF risk by 36% (HR 1.36; 95% CI: 1.30–1.42; *p* < 0.001).	Varies
Hojo et al./non-randomized observational study (2018) [38]	Patients with paroxysmal AF undergoing repeat PVI	100	OSA and CPAP therapy	AF recurrence after PVI	AF recurrence rates: non-OSA (12.1%), treated OSA (9.1%), untreated OSA (8.7%). No significant differences across groups.	Varies
Genuardi et al./retrospective cohort study (2019) [38]	Patients undergoing diagnostic polysomnography (mean age: 51 years)	30,061	Short sleep duration	Prevalence and incidence of AF	Each 1 h reduction in sleep duration increased prevalent AF risk by 17% (OR 1.17; 95% CI: 1.11–1.30) and incident AF risk by 9% (HR 1.09; 95% CI: 1.05–1.13).	Median 4.6 years
Traaen et al./randomized controlled trial (2021) [40]	OSA patients with paroxysmal AF randomized to CPAP or standard care	108	CPAP therapy	AF burden (time in AF)	CPAP did not significantly reduce AF burden (adjusted difference −0.63%; 95% CI: –2.55–1.30%; *p* = 0.52).	5 months
Hunt et al./randomized controlled trial (2022) [39]	OSA patients with paroxysmal AF undergoing PVI	83	CPAP therapy	AF recurrence post-PVI	No significant difference in AF recurrence between CPAP and standard care groups (*p* = 1.0).	12 months
Physical Exertion						
Hegbom et al./randomized controlled trial (2007) [41]	Chronic AF patients aged <75	30	2-month exercise training	HRQoL (SF-36), exercise capacity	Short-term exercise training significantly improved HRQoL (SF-36 physical functioning: 82 ± 14 to 86 ± 10, *p* < 0.01), exercise capacity (41 ± 20% increase, *p* < 0.01), and reduced perceived exertion during exercise (Borg scale −1.4 points, *p* < 0.05). Symptoms (SSCL severity: 12 ± 5 to 10 ± 6, *p* = 0.01) also improved.	2 months
Abdulla et al./systematic review and meta-analysis (2009) [42]	Athletes compared to general population	655 athletes; 895 controls	Athletic training intensity	Incident atrial fibrillation and atrial flutter	Athletes had a significantly higher risk of AF compared to controls (OR 5.29; 95% CI: 3.57–7.85). Risk is associated with increased vagal tone, atrial remodeling, and left atrial enlargement.	Varies
Osbak et al./randomized controlled trial (2011) [43]	Patients with permanent AF	49	Aerobic exercise training	Exercise capacity, 6MWT, quality of life, cardiac output	Exercise training improved exercise capacity (*p* < 0.001), 6MWT (*p* = 0.001), and quality of life (*p* = 0.02) compared to controls. Resting pulse rate decreased (*p* = 0.049). Cardiac output and natriuretic peptides were unchanged.	12 weeks
Pathak et al./observational study (2015) [44]	Obese individuals with atrial fibrillation	308	Cardiorespiratory fitness	Arrhythmia recurrence, cardiovascular health	Higher cardiorespiratory fitness was associated with a lower risk of arrhythmia recurrence (HR 0.87; 95% CI: 0.80–0.93 per 1-MET increase) and improved cardiovascular health outcomes.	49 ± 19 months.
Malmo et al./randomized controlled trial (2016) [45]	Patients with symptomatic, nonpermanent AF	51	Aerobic interval training (AIT)	Time in AF, AF symptoms, cardiac function, quality of life	AIT reduced time in AF (from 8.1% to 4.8%, *p* = 0.001) and improved AF symptom severity, cardiac function, and quality of life compared to control group.	4 weeks
Jin et al./nationwide cohort study (2019) [46]	Korean general population	501,690	Physical activity level	New-onset AF	U-shaped association between physical activity and AF; 500–1000 MET-minutes/week decreased AF risk by 12% (HR: 0.88; 95% CI: 0.80–0.97). Higher physical activity did not provide additional benefits.	Median 4 years
Elliott et al./observational study (UK Biobank) (2020) [47]	402,406 individuals aged 40–69 years from the UK Biobank	402,406	Physical activity levels (MET-minutes/week)	Incident atrial fibrillation (AF) and ventricular arrhythmias	Lower AF risk among physically active participants (HR for 1500 vs. 0 MET-minutes/week: 0.85; 95% CI: 0.74–0.98 in females; HR: 0.90; 95% CI: 0.82–1.0 in males). Lower risk of ventricular arrhythmias (HR: 0.78; 95% CI: 0.64–0.96) observed across a range of 0–2500 MET-minutes/week. Vigorous activity reduced AF risk in females, but no association with bradyarrhythmias.	Over 2.8 million person-years
Mishima et al./systematic review and meta-analysis (2021) [49]	Individuals achieving guideline-recommended PA levels	1,464,539	Physical activity vs. inactivity	Incident of atrial fibrillation	Guideline-recommended PA reduced AF risk (HR 0.94; 95% CI: 0.90–0.97). Dose–response analysis: benefit observed up to 1900 MET-minutes/week. Beyond 2000 MET-minutes/week, benefit is less clear.	Median 4 years
Khurshid et al./prospective cohort study (2021) [48]	Adults without prevalent AF	93,669	Accelerometer-derived activity	Incident atrial fibrillation and stroke	Guideline-adherent activity reduced AF risk (HR 0.82; 95% CI: 0.75–0.89) and stroke risk (HR 0.76; 95% CI: 0.64–0.90). Accelerometer-derived activity correlated weakly with self-reported activity (r = 0.16).	Median 5.2 years
Oesterle et al./meta-analysis of randomized controlled trials (2022) [50]	Participants with paroxysmal or persistent AF	1155	Supervised exercise training	AF recurrence, AF burden, quality of life	Supervised exercise reduced AF recurrence (RR 0.77; 95% CI: 0.60–0.99) and improved quality of life and cardiorespiratory fitness. AF burden reduction was observed in studies with continuous monitoring (SMD −0.49; 95% CI: −0.96–−0.01).	Varied
Elliott et al./supervised exercise intervention (2023) [51]	Symptomatic AF patients	120	Exercise regimen (interval + PA)	AF recurrence, symptom severity, fitness	Reduced AF recurrence and symptom severity over 6–12 months, showing significant rhythm control improvements.	6 months
Obesity						
Tedrow et al./prospective cohort study (Women’s Health Study) (2010) [56]	Healthy women with BMI categories tracked longitudinally	34,309	BMI changes	Incident AF	Each 1 kg/m^2^ BMI increase was associated with a 4.7% higher risk of incident AF (HR 1.65 for obesity; 95% CI: 1.36–2.00; *p* < 0.0001).	12.9 ± 1.9 years
Abed et al./single-center, partially blinded RCT (2013) [57]	Overweight and obese ambulatory AF patients (BMI > 27) randomized to weight management vs. general lifestyle advice	150	Weight reduction and cardiometabolic risk factor management	AF symptom burden and severity, cardiac structure	Weight reduction significantly decreased AF symptom burden scores (intervention: −11.8 vs. control: −2.6 points, *p* < 0.001) and symptom severity scores (intervention: −8.4 vs. control: −1.7 points, *p* < 0.001). Improved left atrial area and interventricular septal thickness.	Median 15 months
Pathak et al./long-term observational cohort (LEGACY study) (2015) [59]	Obese patients (BMI ≥27 kg/m^2^) undergoing weight management programs	355	Weight management	AF burden, symptom severity, and sinus rhythm maintenance	Weight loss of ≥10% reduced AF burden significantly and improved sinus rhythm (6x higher probability of arrhythmia-free survival; *p* < 0.001). Weight fluctuation (> 5%) increased AF recurrence risk (HR 2.0, *p* = 0.02).	Varied follow-up duration (48.4 ± 18.2, 46.0 ± 16.7, and 48.3 ±18.4 months)
Jamaly et al./prospective matched cohort study (Swedish Obese Subjects) (2016) [60]	Obese individuals with no prior AF history; bariatric surgery vs. usual care	4021	Bariatric surgery	New-onset AF	29% lower risk of AF (HR 0.71; 95% CI: 0.60–0.83; *p* < 0.001). Benefits more pronounced in younger patients with high diastolic BP.	Median 19 years
Bunch et al./retrospective cohort study (2016) [61]	AF patients undergoing ablation, stratified by BMI categories (≤20, 21–25, 26–30, and >30 kg/m^2^)	1558	BMI categories	AF recurrence, stroke/TIA, and heart failure	AF recurrence increased with BMI category (*p* = 0.02); stroke risk inversely correlated with BMI (*p* = 0.06). Weight loss reduced AF recurrence risk at 3 years, though not statistically significant after adjustment (HR:1.27, *p* = 0.24).	At least 3 years
Mohanty et al./prospective analysis (2017) [62]	Obese patients with longstanding persistent AF undergoing weight loss interventions	90	Weight loss interventions	AF symptom severity and recurrence post-ablation	Significant weight loss improved QoL but did not significantly reduce symptom severity or long-term ablation outcomes.	1 year
Di Benedetto et al./prospective population study (EPIC-Norfolk) (2018) [63]	Healthy men and women stratified by lifestyle factors (BMI, smoking, alcohol, and activity)	21,499	Lifestyle factors and BMI	Incident AF	Overweight/obesity increased AF risk by 43% (HR 1.43; 95% CI: 1.30–1.57; *p* < 0.0001). Combined lifestyle factors predicted a 2.8-fold difference in AF risk (HR 2.82; 95% CI 1.85–4.29; *p* < 0.0001).	Average of 17.1 years
Lynch et al./propensity-matched cohort study (2019) [65]	Obese patients undergoing bariatric surgery vs. controls without surgery	5044 (2522 surgical, 2522 nonsurgical)	Bariatric surgery	AF incidence	Incidence of AF was significantly lower in surgical patients (0.8% vs. 2.9%, *p* = 0.0001).	For nonsurgical: median value [interquartile range]: 8.0 [9.0] For surgical: median value [interquartile range]: 6.2 [9.2]
Donnellan et al./retrospective cohort study (2019) [64]	Morbidly obese AF patients undergoing ablation (BS group vs. non-BS group)	239 (51 BS, 188 non-BS)	Bariatric surgery	AF recurrence after ablation	AF recurrence significantly lower in BS group (20% vs. 61%, *p* < 0.0001). Hazard ratio for AF recurrence is 0.14 (95% CI: 0.05–0.39, *p* = 0.002).	Mean 36 months
Donnellan et al./retrospective cohort study (2020) [66]	Morbidly obese AF patients with BMI ≥ 40 kg/m^2^ undergoing bariatric surgery	220	Bariatric surgery	AF-type reversal	A total of 71% of gastric bypass, 56% sleeve gastrectomy, and 50% gastric banding patients had AF-type reversal (*p* = 0.004). Significant reduction in BMI, CRP, NT-proBNP, and systolic BP.	Varies
Chokesuwattanaskul et al./systematic review and meta-analysis (2020) [68]	Obese patients undergoing bariatric surgery	7681	Bariatric surgery	AF incidence	Bariatric surgery associated with a 58% reduction in AF risk (OR 0.42; 95% CI: 0.22–0.83; *p* < 0.001).	Median 7.9 years
Srivatsa et al./retrospective cohort study (BLOC-AF) (2020) [67]	Obese patients undergoing bariatric surgery vs. controls undergoing abdominal surgery	4743 (1581 BAS, 3162 controls)	Bariatric surgery	AF hospitalization	29% lower risk of AF hospitalization (HR 0.71; 95% CI: 0.54–0.93; *p* = 0.01) and HF (HR 0.74; 95% CI: 0.60–0.91; *p* = 0.005). Increased risk of GIB (HR 2.1; 95% CI: 1.54–2.95; *p* < 0.0001).	66 months
Höskuldsdóttir et al./nationwide observational cohort study (2021) [69]	Obese individuals with T2DM undergoing bariatric surgery vs. matched controls	10,642 (5321 surgical, 5321 nonsurgical)	Bariatric surgery	Hospitalization for AF	41% lower AF risk in bariatric surgery group (HR 0.59; 95% CI: 0.44–0.78; *p* < 0.001).	Mean 4.5 years
Hypertension						
Schmieder et al./randomized controlled trial (VALUE) (2007) [76]	Hypertensive patients	15,245	Valsartan vs. amlodipine	New-onset atrial fibrillation	Valsartan reduced AF risk compared to amlodipine by 16% (HR 0.84; 95% CI: 0.71–0.99; *p* = 0.045).	6 years ± 0.3 years
Schaer et al./nested case–control analysis (2010) [77]	Hypertensive patients aged 20–79 years from the UK-based General Practice Research Database (GPRD)	4661 cases with atrial fibrillation and 18,642 matched controls	Antihypertensive drug classes (ACE inhibitors, ARBs, β-blockers, calcium-channel blockers)	Risk of incident atrial fibrillation	ACE inhibitors reduced AF risk by 25% (OR 0.75; 95% CI: 0.65–0.87), ARBs reduced AF risk by 29% (OR 0.71; 95% CI: 0.57–0.89), and β-blockers reduced AF risk by 22% (OR 0.78; 95% CI: 0.67–0.92).	Analysis covered data from January 1998 to spring 2008
Schneider et al./meta-analysis of randomized controlled trials (2010) [83]	Hypertensive and other cardiovascular patients	87,048	RAS inhibition	Primary and secondary prevention of AF	RAS inhibition reduced AF risk by 33% (OR 0.67; 95% CI: 0.57–0.78; *p* < 0.00001), with a stronger effect in secondary prevention (OR 0.55; 95% CI: 0.34–0.89).	Varies
Okin et al./retrospective analysis (2015) [78]	Hypertensive patients	8831	Systolic blood pressure levels	New-onset atrial fibrillation	SBP <130 mmHg reduced AF risk by 40% (HR 0.60; 95% CI: 0.45–0.82; *p* < 0.001).	4.6 ± 1.1 years
Chen et al./randomized controlled trial (ACCORD) (2016) [79]	Hypertensive diabetics	3087	Intensive BP control	Atrial fibrillation-related endpoints	Intensive BP control reduced *p*-wave indices and AF-related events (*p* = 0.031).	Mean 4.4 years
Dewland et al./randomized controlled trial (ALLHAT) (2017) [80]	Hypertensive patients	14,837	Lisinopril vs. chlorthalidone	New-onset atrial fibrillation	No significant difference between lisinopril and chlorthalidone in AF reduction (HR 1.04; 95% CI: 0.94–1.15; *p* = 0.46).	7.5 ± 3.2 years
Neefs et al./systematic review and meta-analysis (2017) [82]	Hypertensive patients	5332	Mineralocorticoid receptor antagonists	New-onset atrial fibrillation	MRAs reduced AF incidence significantly (RR 0.78; 95% CI: 0.67–0.90; *p* = 0.002).	Varies
Parkash et al./randomized, open-label clinical trial (SMAC-AF) (2017) [85]	Patients with atrial fibrillation undergoing catheter ablation	184 (92 aggressive BP, 92 standard BP)	Aggressive vs. standard BP lowering	Recurrent symptomatic atrial fibrillation/atrial flutter/atrial tachycardia	No significant reduction in atrial arrhythmia recurrence (HR = 0.94; 95% CI: 0.65–1.38; *p* = 0.763). A subgroup aged ≥ 61 showed a lower event rate (HR = 0.58; 95% CI: 0.34–0.97; *p* = 0.013). Increased adverse events in aggressive BP group.	Median 14 months
Larstorp et al./randomized controlled trial (LIFE) (2019) [75]	Hypertensive patients with isolated systolic hypertension	1248	SBP reduction	New-onset atrial fibrillation	Reduction in in-treatment SBP associated with 17% risk reduction for new-onset AF per 10 mmHg decrease (HR 0.83; 95% CI: 0.73–0.95; *p* = 0.008).	4.8 ± 0.9 years
Soliman et al./randomized controlled trial (SPRINT) (2020) [81]	Hypertensive patients	8022	Intensive vs. standard BP lowering	New-onset atrial fibrillation	Intensive BP control reduced AF risk by 26% (HR 0.74; 95% CI: 0.56–0.98; *p* = 0.037).	Up to 5.2 years
Pinho-Gomez et al./meta-analysis of randomized controlled trials (2021) [84]	Hypertensive patients	188,570	Blood-pressure-lowering intensity	New-onset atrial fibrillation	BP lowering reduced AF risk across studies by 18% (OR 0.82; 95% CI: 0.73–0.93; *p* = 0.002).	Varies
Smoking/Tobacco Use						
Fukamizu et al./observational study (2010) [90]	Patients undergoing PV isolation	59	Smoking status	AF recurrence	Smoker group had 3.19× higher risk of AF recurrence post-ablation compared to non-smokers (RR 3.19; 95% CI: 1.23–8.27; *p* = 0.017).	306 ± 95 days
Chamberlain et al./prospective cohort study (ARIC Study) (2011) [91]	Participants from ARIC study in the USA	15,792	Cigarette smoking (current/former)	Incident atrial fibrillation	Current smokers had 2.05x higher risk of AF (HR 2.05; 95% CI: 1.71–2.47), and former smokers had 1.32× higher risk (HR 1.32; 95% CI: 1.10–1.57).	Mean 13.1 years
Albertsen et al./cohort study (DCH Study) (2014) [92]	Patients with AF in Denmark	3161	Smoking habits	Thromboembolism or death	Heavy smokers (>25 g/day) had 3.64× and 2.17× higher risk of thromboembolism or death in women and men, respectively, compared to non-smokers (adjusted HRs).	Median 4.9 years
Cheng et al./retrospective analysis (2018) [94]	Patients with persistent AF undergoing catheter ablation	201	Smoking (current vs. never)	AF recurrence	Smokers with RA-NPV triggers had worse outcomes post-ablation compared to non-smokers (*p* < 0.05).	31 ± 25 months
Aune et al./systematic review and meta-analysis (2018) [93]	Prospective cohort and nested case–control studies	29 studies	Smoking intensity (current, former, pack-years)	New-onset atrial fibrillation	Current smokers had 1.32× higher risk of AF (RR 1.32; 95% CI: 1.12–1.56), and former smokers had 1.09× higher risk (RR 1.09; 95% CI: 1.00–1.18).	Varies
Choi et al./retrospective cohort study (2020) [95]	South Korean male AF patients	2372	Smoking cessation after AF diagnosis	Cardiovascular disease	Quitters had 35% reduced CVD risk (HR 0.65; 95% CI: 0.44–0.97) compared to continual smokers.	Up to 12 years
Lin et al./large longitudinal cohort study (2022) [96]	California residents with healthcare encounters	23,561,884	Cannabis, cocaine, methamphetamine, and opiate use	Incidence of AF	Cannabis use increased AF risk by 35% (HR 1.35; 95% CI: 1.30–1.40) and methamphetamine use by 86% (HR 1.86; 95% CI: 1.81–1.92).	N/A
Heart Failure						
Olsson et al./subgroup analysis of RCT (CHARM program) (2006) [104]	Patients with chronic HF across EF spectrum	7599	Atrial fibrillation status	Cardiovascular death or HF hospitalization	AF increased risk of adverse outcomes in CHF (HR 1.72 for preserved EF; HR 1.29 for reduced EF). Candesartan showed benefits regardless of rhythm.	Median 37.7 months
ACTIVE investigators/randomized controlled trial (2011) [103]	Patients with atrial fibrillation and additional cardiovascular risk factors	9016	Irbesartan vs. placebo	The primary endpoint was a composite of stroke, myocardial infarction, or death from vascular causes; the secondary endpoint included a composite outcome of hospitalization for heart failure	There was no significant difference in the primary endpoint between irbesartan and placebo (HR 0.99; 95% CI: 0.91–1.08; *p* = 0.85). The secondary composite endpoint, including hospitalization for heart failure, showed no significant difference (HR 0.94; 95% CI: 0.87–1.02; *p* = 0.12). However, irbesartan significantly reduced hospitalization for heart failure alone (HR 0.86; 95% CI: 0.76–0.98; *p* = 0.02).	Median 4.1 years
Zannad et al./randomized controlled trial (EMPHASIS-HF) (2011) [110]	Patients with systolic heart failure and mild symptoms	2737	Eplerenonevs. placebo	Cardiovascular death or HF hospitalization	Eplerenone reduced primary outcome by 37% (HR 0.63; 95% CI: 0.54–0.74; *p* < 0.001). Mortality reduced by 24% (HR 0.76; 95% CI: 0.62–0.93; *p* = 0.008).	Median 21 months
Groenveld et al./randomized controlled trial (RACE II) (2011) [107]	Patients with permanent atrial fibrillation	614	Lenient vs. strict rate control	Quality of life, cardiovascular morbidity, mortality	No significant difference in QOL between lenient and strict rate control. Cardiovascular events and mortality were comparable between groups.	Median 3 years
McMurray et al./randomized controlled trial (PARADIGM-HF) (2014) [111]	Patients with HFrEF	8442	LCZ696 vs. enalapril	Cardiovascular death or HF hospitalization	LCZ696 reduced primary outcome by 20% (HR 0.80; 95% CI: 0.73–0.87; *p* < 0.001). Reduced all-cause mortality by 16% (HR 0.84; 95% CI: 0.76–0.93; *p* < 0.001).	Median 27 months
Kotecha et al./individual patient data meta-analysis (2014) [105]	Patients with HF and atrial fibrillation	18,254	β-blockers vs. placebo	All-cause mortality, cardiovascular hospitalization	β-blockers reduced all-cause mortality in sinus rhythm (HR 0.73; 95% CI: 0.67–0.80; *p* < 0.001) but had no significant benefit in patients with AF (HR 0.97; 95% CI: 0.83–1.14; *p* = 0.73).	Mean 1.5 years
Ziff et al./systematic review and meta-analysis (2015) [108]	Patients treated with digoxin for HF or AF	621,845	Digoxin vs. control	All-cause mortality, hospitalizations	Digoxin associated with neutral mortality effect in RCTs (RR 0.99; 95% CI: 0.93–1.05) and increased mortality in observational studies (RR 1.76; 95% CI: 1.57–1.97). Reduced all-cause hospitalizations across all studies (RR 0.92; 95% CI: 0.89–0.95).	Mean 3.7 years
Solomon et al./subgroup analysis of RCT (TOPCAT) (2015) [114]	Patients with HFpEF	3444	Spironolactone vs. placebo	Cardiovascular death or HF hospitalization	Spironolactone reduced HF hospitalization in lower EF subgroup (HR 0.72; 95% CI: 0.50–1.05). No overall benefit on primary outcome.	Median 3.4 years
Cleland et al./individual patient data meta-analysis of 11 trials (2018) [115]	Patients with HF across EF categories	14,262	β-blockers vs. placebo	All-cause mortality and cardiovascular death	β-blockers reduced all-cause mortality in sinus rhythm (HR 0.73; 95% CI: 0.67–0.79) but showed no significant benefit in patients with AF or EF ≥50%.	Varies
Rienstra et al./randomized controlled trial (RACE 3) (2018) [119]	Patients with early persistent AF and mild-to-moderate HF	245	Targeted vs. conventional therapy	Sinus rhythm maintenance, cardiovascular outcomes	Targeted therapy improved sinus rhythm maintenance at 1 year (OR 1.765; 95% CI: 1.021–3.051; *p* = 0.042). No difference in cardiovascular outcomes at 5 years.	1 year
Lund et al./subgroup analysis of RCT (CHARM-HF) (2018) [113]	Patients with HF with mid-range ejection fraction	7598	Candesartanvs. placebo	Cardiovascular death or HF hospitalization	Candesartan reduced primary outcome in HFmrEF (HR 0.76; 95% CI: 0.61–0.96; *p* = 0.02). No benefit observed in HFpEF subgroup.	Mean 2.9 years
Kotecha et al./randomized controlled trial (RATE-AF) (2020) [106]	Patients with permanent AF and symptoms of HF	160	Digoxin vs. bisoprolol	Quality of life, symptoms, NT-proBNP, adverse events	Digoxin showed significantly better symptom control (53% vs. 9% two-class EHRA improvement, *p* < 0.001) and fewer adverse events (25% vs. 64%, *p* < 0.001) in 6 and 12 months. NT-proBNP levels lower with digoxin at 12 months (*p* = 0.005).	12 months
Pandey et al./systematic review and meta-analysis (2021) [19]	Patients treated with SGLT inhibitors	75,279	SGLT inhibitors vs. control	Atrial fibrillation, HF hospitalization, cardiovascular death	SGLT inhibitors reduced serious AF events (RR 0.75; 95% CI 0.66–0.86) and HF hospitalization or cardiovascular death in patients with AF (HR 0.70; 95% CI 0.57–0.85).	Varies
Bhatt et al./randomized controlled trial (SOLOIST-WHF) (2021) [117]	Diabetic patients with recent HF hospitalization	1222	Sotagliflozin vs. placebo	Composite of CV death, HF hospitalization, urgent HF visits	Sotagliflozin reduced event rates (HR 0.67; 95% CI: 0.52–0.85; *p* < 0.001).	Median 9 months
Anker et al./randomized controlled trial (EMPEROR-Preserved) (2021) [116]	Patients with HF and preserved EF	5988	Empagliflozin vs. placebo	Composite of CV death or HF hospitalization	Empagliflozin reduced primary outcome (HR 0.79; 95% CI: 0.69–0.90; *p* < 0.001), driven by fewer HF hospitalizations.	Median 26.2 months
Solomon et al./randomized controlled trial (DELIVER) (2022) [118]	Patients with HFpEF or mildly reduced EF	6263	Dapagliflozin vs. Placebo	Cardiovascular death, worsening HF	Dapagliflozin reduced risk of composite endpoint (HR 0.82; 95% CI: 0.73–0.92; *p* < 0.001), including reductions in cardiovascular death and hospitalization for HF.	Median 2.3 years
Nguyen et al./randomized controlled trial (RACE 3 follow-up) (2022) [120]	Patients with early persistent AF and mild-to-moderate HF	216	Targeted vs. conventional therapy	Sinus rhythm maintenance, cardiovascular outcomes	Targeted therapy did not improve sinus rhythm maintenance or cardiovascular outcomes at 5 years compared to conventional therapy (*p* > 0.05).	5 years
Stress and Depression						
Thrall et al./prospective cohort study (2007) [126]	Patients with atrial fibrillation (AF) compared to hypertensive patients in sinus rhythm (control group)	101 AF patients, 97 hypertensive controls	Depression and anxiety (measured by Beck Depression Inventory and State–Trait Anxiety Inventory)	Impact of depression and anxiety on quality of life (QoL) over 6 months	A total of 38% of AF patients had symptoms of depression and trait anxiety which persisted in the 6-month follow-up (36.8%). A total of 28% had state anxiety which persisted in the 6-month follow-up. (33.3%) Depression was the strongest predictor of reduced QoL at 6 months (R^2^ = 0.20), followed by gender and employment status.	6 months
Graff et al./population-based cohort study (2017) [127]	Participants from the Danish National Health Survey	114,337	Perceived stress measured by Cohen’s 10-item Perceived Stress Scale (PSS)	Incident AF	Initially, high perceived stress was associated with a 28% increased risk of AF (95% CI: 12%–46%). After adjusting for comorbidities, socioeconomic status, and lifestyle factors, the association was no longer significant (HR 1.01; 95% CI: 0.88–1.16).	4 years
Chou et al./nationwide nested case–control study (2017) [133]	General population from Taiwan’s National Health Insurance Database	68,972 subjects, including 34,053 cases of new-onset atrial fibrillation (AF) and 34,919 matched controls	Antipsychotic exposure	Incidence of AF	Use of antipsychotics had a 17% increased risk of AF (adjusted OR: 1.17, 95% CI: 1.10–1.26). Risk of AF was increased in a dose-dependent way with antipsychotic usage (*p* < 0.001). Agents with higher muscarinic M2 receptor binding affinity showed a higher incidence of AF.	10 years
Fransson et al./prospective cohort study and meta-analysis (2018) [134]	Swedish working population from the Swedish Longitudinal Occupational Survey of Health (SLOSH)	13,200 participants (5980 men and 7220 women)	Job strain as a measure of work stress	Incident AF	Job strain was associated with an almost 50% increased risk of AF (HR 1.48, 95% CI: 1.00–2.18) after adjusting for age, sex, and education. A meta-analysis of this study with two previous studies yielded a pooled HR of 1.37 (95% CI: 1.13–1.67), indicating enhanced risk for AF.	Median of 5.7 years
Garg et al./prospective cohort study (2019) [123]	Multi-ethnic participants from the MESA (Multi-Ethnic Study of Atherosclerosis) cohort, free of AF at baseline	6644	Depressive symptoms, anger, anxiety, and chronic stress	Incident AF	Depressive symptoms, defined by a CES-D score ≥ 16 or antidepressant use, were associated with a higher risk of AF (HR 1.34, 95% CI: 1.04–1.74 for CES-D ≥ 16; HR 1.36, 95% CI: 1.04–1.77 for antidepressant use).	Median of 13 years
Feng et al./prospective cohort study (2020) [129]	Adults from the HUNT study in Norway	37,402	Symptoms of anxiety and depression measured by the Hospital Anxiety and Depression Scale (HADS)	Incident AF	Mild-to-moderate depression was associated with an increased risk of AF (multivariable-adjusted HR 1.5; 95% CI: 1.2–1.8), while severe depression showed no significant association (multivariable-adjusted HR 0.9; 95% CI: 0.6–1.3).	Median of 8.1 years
Fenger-Grøn et al./nationwide cohort study (2021) [131]	Danish patients with atrial fibrillation (AF)	147,810 patients with incident AF and increased risk status	Presence of bipolar disorder or schizophrenia	Initiation and prevalence of OAT	Both bipolar disorder and schizophrenia were associated with significantly lower rates of OAT initiation within 90 days of AF diagnosis and lower OAT prevalence. Bipolar disorder was linked to a −12.7% lower initiation rate (95% CI: −15.3%–−10.0%) and −11.6% lower prevalence (95% CI: −13.9% –−9.3%). Schizophrenia showed a −24.5% lower initiation rate (95% CI: −28.3%–−20.7%) and −21.6% lower prevalence (95% CI: −24.8%–−18.4%). After adjusting for socioeconomic factors and comorbidities, disparities remained significant, particularly for schizophrenia.	Up to 11 years
Du et al./meta-analysis of cohort studies (2021) [128]	AF patients undergoing catheter ablation from five cohort studies	549	Presence of anxiety	Recurrence of AF after catheter ablation	Anxiety was associated with a higher risk of AF recurrence post-ablation (adjusted RR 2.36; 95% CI: 1.71–3.26; *p* < 0.001).	Average of 9.7 months
Wu et al./meta-analysis and systematic review (2022) [132]	General population across 13 studies	5,329,908 participants	Psychological factors including anxiety, anger, depression, and work stress	Incident AF	Anxiety increased AF incidence by 10% (HR 1.10, 95% CI: 1.02–1.19), anger by 15% (HR 1.15, 95% CI: 1.04–1.26), depression by 25% (HR 1.25, 95% CI: 1.12–1.39), and work stress by 18% (HR 1.18, 95% CI: 1.05–1.32).	3.8 to 23.4 years across studies
Højen et al./nationwide cohort study (2022) [130]	Patients with incident atrial fibrillation (AF) and schizophrenia identified from Danish health registries	673 patients with schizophrenia matched to 3265 patients without schizophrenia	Presence of schizophrenia	Initiation of OAC	OAC initiation was substantially lower in AF patients with schizophrenia (33.7%) compared to those without (54.4%), with an adjusted risk difference of −20.7% (95% CI: −24.7–−16.7). OAC initiation increased over time for both groups, but disparities persisted.	1 year
Kim et al./nationwide cohort study (2022) [124]	South Korean adults from the Korean National Health Insurance Service database	5,031,222	Previous diagnosis of depression and recurrent episodes of depression	New-onset AF	Depression was associated with a 25.1% increased risk of new-onset AF after adjusting for covariates (HR 1.25, 95% CI: 1.22–1.29). Recurrent episodes of depression were linked to an even higher risk (HR 1.32, 95% CI: 1.27–1.37). Younger age and female sex had significant interactions with depression, indicating higher susceptibility in these groups.	10 years (43,115,042 person-years)
Bae et al./nationwide population-based cohort study (2022) [121]	Patients with diabetes mellitus from the Korean National Health Insurance Service database	2,512,690	Presence of mental disorders (depression, insomnia, anxiety, bipolar disorder, schizophrenia)	New-onset AF	Patients with diabetes and mental disorders had a significantly higher risk of developing AF (adjusted HR 1.19; 95% CI: 1.17–1.21) with specific risks for depression (HR 1.15), insomnia (HR 1.15), and anxiety (HR 1.19). Bipolar disorder and schizophrenia were not significantly associated with increased AF risk.	Median of 7.1 years
Ahn et al./nationwide population-based study (2023) [125]	South Korean adults aged 20–39 years from the Korean National Health Insurance Database	6,576,582	Diagnosis of mental disorders (depression, insomnia, anxiety disorder, bipolar disorder, schizophrenia)	New-onset AF	Mental disorders were associated with a significantly higher risk of AF (adjusted HR 1.526; 95% CI: 1.436–1.621). Bipolar disorder or schizophrenia had a 2-fold higher risk of AF, while those with depression, insomnia, and anxiety disorder had a 1.5- to 1.7-fold increased risk compared to individuals without mental disorders.	Varies

N/A = Not Available.
